# Loss-of-function variants in the *KCNQ5* gene are implicated in genetic generalized epilepsies

**DOI:** 10.1016/j.ebiom.2022.104244

**Published:** 2022-09-09

**Authors:** Johanna Krüger, Julian Schubert, Josua Kegele, Audrey Labalme, Miaomiao Mao, Jacqueline Heighway, Guiscard Seebohm, Pu Yan, Mahmoud Koko, Kezban Aslan-Kara, Hande Caglayan, Bernhard J. Steinhoff, Yvonne G. Weber, Pascale Keo-Kosal, Samuel F. Berkovic, Michael S. Hildebrand, Steven Petrou, Roland Krause, Patrick May, Gaetan Lesca, Snezana Maljevic, Holger Lerche

**Affiliations:** a Department of Neurology and Epileptology, Hertie Institute for Clinical Brain Research, University of Tübingen, Otfried-Müller-Straße 27, 72076 Tübingen, Germany; b Service de Génétique, Hospices Civils de Lyon, Groupement Hospitalier Est, 59 Boulevard Pine, 69677 Bron, France; c Florey Institute of Neuroscience and Mental Health, University of Melbourne, 30 Royal Parade, Parkville 3052, VIC, Australia; d Institute for Genetics of Heart Diseases (IfGH), Department of Cardiovascular Medicine, University Hospital Münster, Domagkstraße 3, 48149 Münster, Germany; e Çukurova University, Faculty of Medicine, Department of Neurology, Balcali 01790, Saricam/Adana, Turkey; f Department of Molecular Biology and Genetics, Boğaziçi University, Bebek 34342, Istanbul, Turkey; g Kork Epilepsy Center, Landstraße 1, 77694 Kehl-Kork, Germany; h Department of Epileptology and Neurology, University of Aachen, Pauwelsstraße 30, 52074 Aachen, Germany; i Epileptology, Sleep Disorders and Functional Pediatric Neurology, Member of ERN-EpiCARE; HFME, Hospices Civils de Lyon, 59 Boulevard Pinel, 69500 Bron, France; j Epilepsy Research Centre, Department of Medicine, Austin Health, The University of Melbourne, 245 Burgundy Street, Heidelberg 3084,VIC, Australia; k Luxembourg Centre for Systems Biomedicine, University of Luxembourg, 6 Avenue du Swing, Belvaux 4367, Luxembourg

**Keywords:** *KCNQ5*, Genetic generalized epilepsy, Exome sequencing, Loss-of-function, Patch-clamp

## Abstract

**Background:**

*De novo* missense variants in *KCNQ5*, encoding the voltage-gated K^+^ channel K_V_7.5, have been described to cause developmental and epileptic encephalopathy (DEE) or intellectual disability (ID). We set out to identify disease-related *KCNQ5* variants in genetic generalized epilepsy (GGE) and their underlying mechanisms.

**Methods:**

1292 families with GGE were studied by next-generation sequencing. Whole-cell patch-clamp recordings, biotinylation and phospholipid overlay assays were performed in mammalian cells combined with homology modelling.

**Findings:**

We identified three deleterious heterozygous missense variants, one truncation and one splice site alteration in five independent families with GGE with predominant absence seizures; two variants were also associated with mild to moderate ID. All missense variants displayed a strongly decreased current density indicating a loss-of-function (LOF). When mutant channels were co-expressed with wild-type (WT) K_V_7.5 or K_V_7.5 and K_V_7.3 channels, three variants also revealed a significant dominant-negative effect on WT channels. Other gating parameters were unchanged. Biotinylation assays indicated a normal surface expression of the variants. The R359C variant altered PI(4,5)P_2_-interaction.

**Interpretation:**

Our study identified deleterious *KCNQ5* variants in GGE, partially combined with mild to moderate ID. The disease mechanism is a LOF partially with dominant-negative effects through functional deficits. LOF of K_V_7.5 channels will reduce the M-current, likely resulting in increased excitability of K_V_7.5-expressing neurons. Further studies on network level are necessary to understand which circuits are affected and how this induces generalized seizures.

**Funding:**

DFG/FNR Research Unit FOR-2715 (Germany/Luxemburg), BMBF rare disease network Treat-ION (Germany), foundation ‘no epilep’ (Germany).

**Research in context**

**Evidence before this study**

Recently, four variants in *KCNQ5* have been identified in patients with ID and/or DEE and were functionally characterised. Furthermore, a more recent case report has found an intragenic duplication of this gene resulting in a haploinsufficiency in a patient with ID and absence seizures. No other pathogenic variants have been reported, even though the K_V_7 channel family in general has been associated with many diseases. We screened multiple cohorts of individuals with GGE to identify ultra-rare variants and expand the known phenotypic spectrum of this gene.

**Added value of this study**

We found that pathogenic variants in *KCNQ5* are very rare among GGE patients, yet they are enriched as compared to control cohorts. Functional analysis via patch-clamp recordings in heterologous expression systems revealed a LOF in 5/7 variants, two were found to be benign. Of these five variants three were found to be dominant negative when co-expressed with the WT and K_V_7.3-WT subunits. Biotinylation assays showed that all variants were inserted in the membrane indicating that the LOF is caused by an opening inability of the channel, which was tested using the most severe variant that showed a complete, dominant negative LOF. This variant (p.R359C) showed reduced PIP_2_ binding ability in overlay assays and a homology model indicating that this variant is unable to properly interact with the PIP_2_ resulting in an inability to open.

**Implications of all the available evidence**

This study expands the phenotypic spectrum of patients with pathogenic *KCNQ5* variants, although variants in individuals with GGE seem to be very rare. All pathogenic variants cause a LOF and thus induce at least a haploinsufficiency. Dominant-negative variants were found to decrease the probability of channel opening. This is of importance as it opens new treatment opportunities using K_V_7 channel openers such as ezogabine for these individuals when they become pharmaco-resistant.

Alt-text: Unlabelled box

## Introduction

*KCNQ5* is one of five members of the highly conserved *KCNQ* gene family, which encodes the α-subunits of the M-type, voltage-gated delayed rectifier potassium channels K_v_7.1-7.5.^1^^,^^2^ Four subunits can form a functional channel as either homo- or heterotetramers. Heteromeric channels containing K_v_7.3 subunits together with K_V_7.5 or K_V_7.2 have been shown to yield larger K^+^ currents than each of the subunits alone.^1^^,^^2^^,^^3^ *KCNQ1* is mainly expressed in cardiac muscle and the cochlea,^4^ *KCNQ2* and *-3* are mainly expressed throughout the central nervous system (CNS) and peripheral nervous system (PNS),^5^ *KCNQ4* is expressed in sensory outer hair cells^6^ and *KCNQ5* is expressed in the brain, skeletal muscle and blood vessels.^1^^,^^2^^,^^7^ In addition to heteromerization, native K_V_7 currents, particularly those of K_V_7.1, can be influenced by assembly with *KCNE* subunits and variants in either one of them can cause cardiac arrhythmia or deafness.^4^^,^^8^ Due to its very restricted expression, *KCNQ4* is a key contributor to the auditory system and its dysfunction has been associated with non-syndromic dominant deafness.^6^

The other three family members, *KCNQ2, -3* and *-5*, are important regulators of the neuronal M-current within the nervous system that very effectively controls neuronal firing.^9^ In particular, dominant negative K_v_7.5 channel expression has been found to decrease the medium and slow afterhyperpolarization currents in the CA3 region of the hippocampus in a mouse model.^10^ Moreover, this model unravelled the role of *KCNQ5* in attenuating synaptic inhibition and modifying hippocampal network synchronization.^11^ Upon application of muscarinic receptor agonists, such as acetylcholine, a signalling cascade is triggered that causes depletion of phosphatidylinositol-4,5-bisphosphate (PI(4,5)P_2_) from the membrane, which forces K_v_7 channels to close reducing the M-current and resulting in increased neuronal firing.^12^

Loss-of-function (LOF) variants in *KCNQ2* and *KCNQ3* were first identified as the cause of epileptic seizures in patients with benign familial neonatal epilepsy (BFNE) and later in developmental and epileptic encephalopathies (DEE).^13^^,^^14^^,^^15^ More recently, *de novo* heterozygous missense and truncating variants in *KCNQ5* in individuals with intellectual disability (ID) alone or with DEE have been described.^16^^,^^17^^,^^18^ Additionally, an individual presenting with absence seizures in adolescence, migraine and mild ID has recently been identified with an intragenic duplication of *KCNQ5* most likely causing haploinsufficiency by skipping exons 2-11 and resulting in a premature stop codon.^19^

Here, we analysed different cohorts of GGE patients to identify causative variants in *KCNQ5*, studied the phenotypes of affected individuals and co-segregation of the detected variants. Electrophysiological and biochemical characterization of variants contributing to the disease was performed by expression of mutant K_v_7.5 subunits in Chinese hamster ovary (CHO) cells and using whole cell patch-clamping, biotinylation and phospholipid overlay assays.

## Subjects/materials and methods

### Study participants

This study was approved by the local institutional review boards of the participating centres. The patients or their relatives gave their written informed consent. In total, 10 individuals from 5 families were ascertained from three different cohorts. Individuals 1, 2, 3, 4, 6 and 7 were ascertained from the EuroEPINOMICS-CoGIE study, while individual 5 was ascertained from the Epi25 study (Tuebingen subcohort), individuals 8 and 9 were analysed using a gene panel in a French cohort, and individual 10 was ascertained from the EPGP/Epi4K study. Two additional independent individuals from the latter cohort carried benign *KCNQ5* variants and are not considered further in our study (compare first paragraph of the results section). Medical and family histories, neurological examination, brain imaging and EEG findings were analysed. Seizure types were classified according to the latest International League Against Epilepsy classification.^20^ Blood samples were taken from available family members and DNA was extracted by standard procedures.

### Genetic analysis

DNA from individuals from all cohorts were whole exome sequenced, whereas individuals 8 and 9 were analysed in an epilepsy gene panel. Validation of discovered *KCNQ5* variants was performed via Sanger sequencing with the exception of family 3 due to insufficient amount of DNA. Splice site variants were further investigated by RNA extraction from patient blood (PAXgene Blood RNA System, BD), and subsequent cDNA amplification and sequencing.

### Variant Interpretation

*KCNQ5* variants were considered putatively disease-relevant if 3/4 of the following criteria were met (1) likely functional effect (protein-truncating variant, inframe-deletion or a missense variant with at least 4 pathogenic predictions), (2) minor allele frequency (MAF) of 0 in the European populations of the 1000 genomes (http://www.1000genomes.org), the Exome Variant Server (EVS; http://evs.gs.washington.edu) and in the Genome Aggregation Database (gnomAD; http://gnomad.broadinstitute.org), (3) confirmed in all affected family members, and (4) the variant demonstrated an abnormal effect in electrophysiological recordings or analysis of splicing. The variants were interpreted according to the American College of Medical Genetics and Genomics standards and guidelines for the interpretation of sequence variants.^21^

### Testing for variant enrichment

To estimate the burden of qualifying variants in *KCNQ5* in GGE cases vs. controls, we extended the rare-variant association analysis performed by Epi4K/EPGP^22^ using additional cohorts of unrelated GGE patients and matched controls, all of European ancestry. A combined non-overlapping set of 4,418 GGE cases and 7,727 controls from these previously published cohorts was investigated using the Cochran-Mantel-Haenszel exact test as follows: 1) 640 cases and 3,877 controls from the Epi4K/EPGP study,^22^ 2) 874 cases and 2,177 matched controls from EuroEPINOMICS-CoGIE, EpiPGX & CENet consortia studies,^23^^,^^24^ 3) 2,904 cases and 1,763 matched controls from the Epi25 Collaborative study.^25^ Qualifying variants were defined consistently across cohorts using the criteria previously defined by Epi4K/EPGP.^22^

### Functional analysis

#### Mutagenesis

pcDNA3.1-P2A-eGFP and pcDNA3.1-P2A-tagRFP vectors containing the human K_v_7.5-subunit cDNA (NM_019842.3) were purchased from GeneScript (Netherlands). Site-directed mutagenesis was performed using PCR with Pfu polymerase (Promega, Germany). The inserts were sequenced to confirm the introduction of the point mutations and to exclude additional alterations. A pcDNA3-PIP5Kγ (ΝΜ_001146687.2) plasmid was kindly gifted by Alvaro Villarroel (Instituto Biofisika, University of Basque Country, Leioa, Spain). pDrVSP-IRES2-EGFP was a kind gift from Yasushi Okamura (Addgene plasmid # 80333; http://n2t.net/addgene:80333; RRID:Addgene_80333).

#### Transfection and expression in CHO cells

CHO-K1 cells were cultured at 37 °C in a 5% CO_2_ humidified atmosphere and grown in Ham's F12 containing 2mM glutamine (Gibco), 10% (v/v) fetal calf serum (PAN Biotech; Tuebingen cohort) or a 1:1 Dulbecco's modified Eagle medium (DMEM)/Ham'sF12 mix including 10% (v/v) fetal calf serum and 1% Penicillin-Streptomycin (Gibco; + antibiotics cohort) in 3·5 cm plastic dishes. Transfection was performed 24-48 h prior to electrophysiological recordings with Lipofectamine 3000 (Invitrogen) following the manufacturer's protocol using 2-2·5 µg DNA and an additional 0·25 µg of eGFP DNA for the truncation variant (p.A301Gfs*64) lacking the fluorescent marker due to the early stop codon. For co-transfection with WT subunits the same protocol was applied using 2–2·5 µg of DNA in total in a molar ratio of 1:1. For the WT controls only 1 µg of DNA (P2A-tagRFP construct) was used. For co-expression of K_v_7.3, a CHO-K1 line stably expressing K_V_7.3 channels was used and transiently transfected as mentioned above. This cell line was created by Lenti-viral transduction of CHOs using a pLenti4/TO/V5-DEST gateway vector carrying human K_v_7.3 WT cDNA. Zeocin was used for selection of transduced cells and removed 72 h prior to recordings. Expression of the introduced K_v_7.3 WT was further confirmed by Western blot. For PIP_2_ depletion or augmentation, cells were co-transfected with 2 µg of *KCNQ5*-WT or -R359C cDNA and 2 µg of either the DrVSP or PIP5K plasmid.

#### Electrophysiology

Standard whole-cell patch clamp recordings were performed using an Axopatch 200B or Multiclamp 700B amplifier, a Digidata 1320A, 1440A or 1550B digitizer (Axon Instruments), and pCLAMP 8, 10.4 or 11.1 data acquisition software (Molecular Devices). Leakage and capacitive currents were automatically subtracted using a pre-pulse protocol (-P/4). Cells were held at -80 mV in whole-cell configuration for 2 min prior to recording and series resistance was compensated (at approx. 85%) and monitored regularly. Currents were filtered at 1 kHz and digitized at 5 kHz. The bath solution contained (in mM): 138 NaCl, 2 CaCl2, 5·4 KCl, 1 MgCl2, 10 glucose and 10 (4-(2-hydroxyethyl)-1-piperazineethanesulphonic acid (HEPES) (pH 7·4 adjusted with NaOH). Borosilicate glass pipettes had a final tip resistance of 1.5-3.5 MΩ and were filled with pipette solution containing (in mM): 140 KCl, 2 MgCl2, 10 EGTA, 10 HEPES, 5 K_2_ATP (pH 7·4 adjusted with KOH).^26^ All recordings were performed at room temperature of 21- 23 °C (RT).

Cells were visualized using an inverted microscope (Axio-Vert.A1, Zeiss or Nikon Eclipse). In case of single plasmid transfections only green (eGFP) or red (tagRFP for the WT in heteromeric expression experiments) fluorescent cells were selected for electrophysiological recordings 24–48 h after transfection, whereas in co-transfection recordings, cells were selected that showed an approximately equal amount of both, red and green fluorescence.

#### Patch clamp protocols and data analysis

K^+^ currents were induced by depolarizing the membrane from a holding potential of -80 mV to +60 mV in 10 mV steps for 2 s. Subsequently, a shorter hyperpolarizing pulse was elicited to −120 mV for 0·5 s to obtain tail currents. Current amplitudes were calculated from the mean steady-state current for the last 0·5 s of the first step depolarization. Current densities (pA/pF) were obtained by normalizing the current amplitudes to the cell membrane capacitance. The activation curve was determined by plotting the normalized tail (I_tail_) current against the step potential (V_s_). A Boltzmann function, I_tail_ = 1/(1 + exp[(V_0.5_ – V_s_)/k]), where V_0.5_ is the voltage of half-maximal activation and k is the slope factor, was fit to the data points. In experiments using voltage-sensing phosphatase, a step from –80 mV to 0 mV was applied for 2 s followed by a step to +100 mV for 0.2-2.0 s to activate the phosphatase before decreasing membrane voltage back to 0 mV for 25 s. Data from recordings using voltage-sensing phosphatase were analysed by normalizing the negative peak to the mean current before the step to +100 mV for each recording and results were fit with a sigmoidal function. Furthermore, the recovery of current over time was analysed by normalizing the current at each second after the step to the mean current prior to the step. Clampfit software of pClamp10.7 (Axon Instruments), Microsoft Excel (Microsoft Corporation, Redmond, WA, USA) or GraphPad software (GraphPad Prism 8, San Diego, CA, USA) were used for data and statistical analysis. All data is shown as mean ± SEM. All data were tested for normal distribution. One-way ANOVA with Dunnett's *post hoc* test or Student's unpaired t-test were used to evaluate statistical significance of normally distributed data. If the data was not normally distributed, a Kruskal-Wallis test was performed followed by a Benjamini, Krieger, and Yekutieli test. For all statistical tests p<0·05 was considered significant. Scatter plots show single cell values, median and interquartile range.

#### Western blot analysis

CHO cells were lysed 24 h after transfection with either wildtype *KCNQ5*, one of the mutant cDNAs or water (mock) using the following buffer (in mM): 20 Tris (pH 7.5), 150 NaCl, 1 EDTA, 1 EGTA, 2·5 Napyrophosphate, 1 β-glycerolphosphate, 1 sodium-orthovanadate, 10 DTT, 1% Triton and 1x cOmplete protease inhibitor cocktail solution (Roche). Total protein concentration was measured via Bradford assay. 8 % polyacrylamid gels were loaded with 20 µg of total protein per lane to separate these by sodium dodecyl sulfate-polyacrylamide gel electrophoresis (SDS PAGE). After transferring the proteins onto a nitrocellulose membrane (Whatman) via electrophoresis at 4 °C in Towbin buffer (25 mM Tris, 192 mM glycine, pH 8·3, 10% (v/v) methanol), the blots were blocked in 5% non-fat dry milk powder in phosphate-buffered saline with 1% Tween (PBST) for 1 h at RT. Subsequently, membranes were probed with a polyclonal rabbit primary antibody against K_V_7.5 (ABN1372, Millipore) at 1:7,500 and a monoclonal mouse primary antibody against actin (A5441, Sigma-Aldrich) at 1:30,000 overnight at 4 °C. If these experiments were conducted with the stable K_V_7.3 cell line, an additional primary antibody against K_V_7.3 (APC-051, Alomone labs) was used. Following this, the membranes were washed and shaken in PBST thrice and then re-probed with a secondary goat anti-rabbit IgG-HRP-conjugated antibody (172-1019, Bio-Rad) at 1:10,000 or secondary goat anti-mouse IgG-HRP-conjugated antibody (172-1011, Bio-Rad) at 1:10,000, respectively, for 1 h at RT. After three more washing steps in PBST, detection was performed via enhanced chemiluminescence (ECL; Amersham, Cytiva). Quantitative analysis was performed using ImageJ 1.52r.^27^

#### Biotinylation assay

For isolation of membrane proteins, the Pierce Cell Surface Protein Biotinylation and Isolation Kit (ThermoFisher) was used according to the manufacturer's protocol. In brief, cells were cultured for 48 h and transfected with either the WT or one of the mutant plasmids. Cells were biotinylated, lysed and isolated by binding to probed agarose beads 24 h after transfection. After elution, the proteins were prepared for Western blot analysis. Western blots were performed and analysed as described above. A monoclonal mouse anti-actin primary antibody (1:30,000, A5441, Sigma-Aldrich) was used as control.

#### Protein-phospholipid overlay assay

PIP strips (Molecular Probes) were used as described in the manufacturer's protocol. Concisely, cells were cultured and transfected as described above and lysed 24 h after transfection. PIP strips were blocked in 3% fatty-acid free bovine serum albumin (BSA) in Tris-buffered saline with 1 % Tween (TBST) for 1 h at RT. Next, membranes were incubated at 4 °C overnight in TBST+3% BSA and a final protein concentration of 1 µg/ml. Membranes were washed and treated, developed and analysed the same way as Western blots described above.

#### Molecular modelling

Molecular modelling was performed using YASARA Structure, ver. 21.12.19 and OriginPro 2022 for data analysis. The K_V_7.5 homology model was calculated based on the closely related K_V_7.4 structure in complex with PI(4,5)P_2_ 7VNP.pdb.^28^ Five alternative alignments of K_V_7.4 vs. -7.5 were generated with a maximum allowed (PSI-)BLAST E-value to consider a template (EValue Max) of 0.1. PSI-BLAST was applied to create a target sequence profile and feeding it to the PSI-Pred secondary structure prediction algorithm.^29^ Five K_V_7.5 homology models were calculated based on the alignments. The homology modelling parameters were: modelling speed (slow = best): Slow; maximum oligomerization state (OligoState): 4 (tetrameric); maximum number of conformations tried per loop (LoopSamples): 50; maximum number of residues added to the termini (TermExtension): 10. Using YASARA Structure a consensus K_V_7.5 homology model covering K_V_7.5 residues 92-586 was generated. This model was employed for further K_V_7.5 control simulation 1. To generate two slightly different start conformations, the model was energy minimized a second time and the resultant slightly different model was used for control simulation 2. To generate a K_V_7.5 structure complexed to PI(4,5)P_2_, the PI(4,5)P_2_ molecules present in the template K_V_7.4 structure 7VNP.pdb were positioned in virtually identical position into the two K_V_7.5 consensus homology models. Subsequent energy minimization was applied to generate the K_V_7.5-PI(4,5)P_2_ models used for further simulations in presence of PIP_2_. The energy minimization procedure included an initial local steepest decent minimization without electrostatics to remove bumps followed by a simulated annealing minimization to reduce the energy of the K_V_7.5-PIP_2_ complex. K_V_7.5-R359C and K_V_7.5-R359C-PIP_2_ complexes were generated by swapping arginine 359 to cysteine in the individual models followed by energy minimizations. Molecular dynamics simulation of the WT or mutant K_V_7.5 or K_V_7.5-PIP_2_ membrane proteins were run using force field AMBER14. Simulation temperature was set to 298 K and pressure was set to 1 bar at an electrostatics cutoff=8. The ion concentration in the solute was 0.9% NaCl (mass fraction) at pH =7.4. The channel models were embedded in phosphatidyl-ethanolamine (PEA) membranes in a square shaped simulation box that was 20 Å larger than the protein (periodic boundary). Membrane insertion of channels was performed using YASARA Structure standard procedure that scans the protein for putative transmembrane segments and positions the protein accordingly into the membrane. Membrane density of “1” is achieved by firstly insertion of a xy-directional-shrunken model into a central hole pinched in the membrane and subsequent extension plus MD simulation of the protein-membrane to normal size. MD simulation on the resultant system was initiated by an equilibration period of 250 picoseconds. During this initial equilibration phase, the membrane is artificially stabilized to allow for realistic repacking and cover the solute, while solvent molecules H_2_O, Na^+^ and Cl^−^ are kept outside of the membrane region. The following MD simulation was computed as all-atoms-mobile simulation. All eight simulations were run for 100 nsec. The simulations were run on a 32-core AMD Ryzen Threadripper 2990WX computer equipped with 4 Geforce 2080 TI graphic cards installed. As the modelling systems were relatively extensive (about 330.000 atoms) the individual simulation took about 6-8 weeks each. Root mean square deviation (RMSD), RMSF and Cα-cross-distances were calculated with provided YASARA macros md_analyze.mcr or manually. Where relevant, significance of mean differences for simulation data was tested by paired Student's t-test conditions within simulation 1 or simulation 2 indicated by * for *p* < 0.05.

### Role of funding source

The funders had no role in study design, data collection, analyses, and interpretation of the data or in the writing of this publication.

## Results

### Genetic Screening of GGE Cohorts

**Figure 1:**
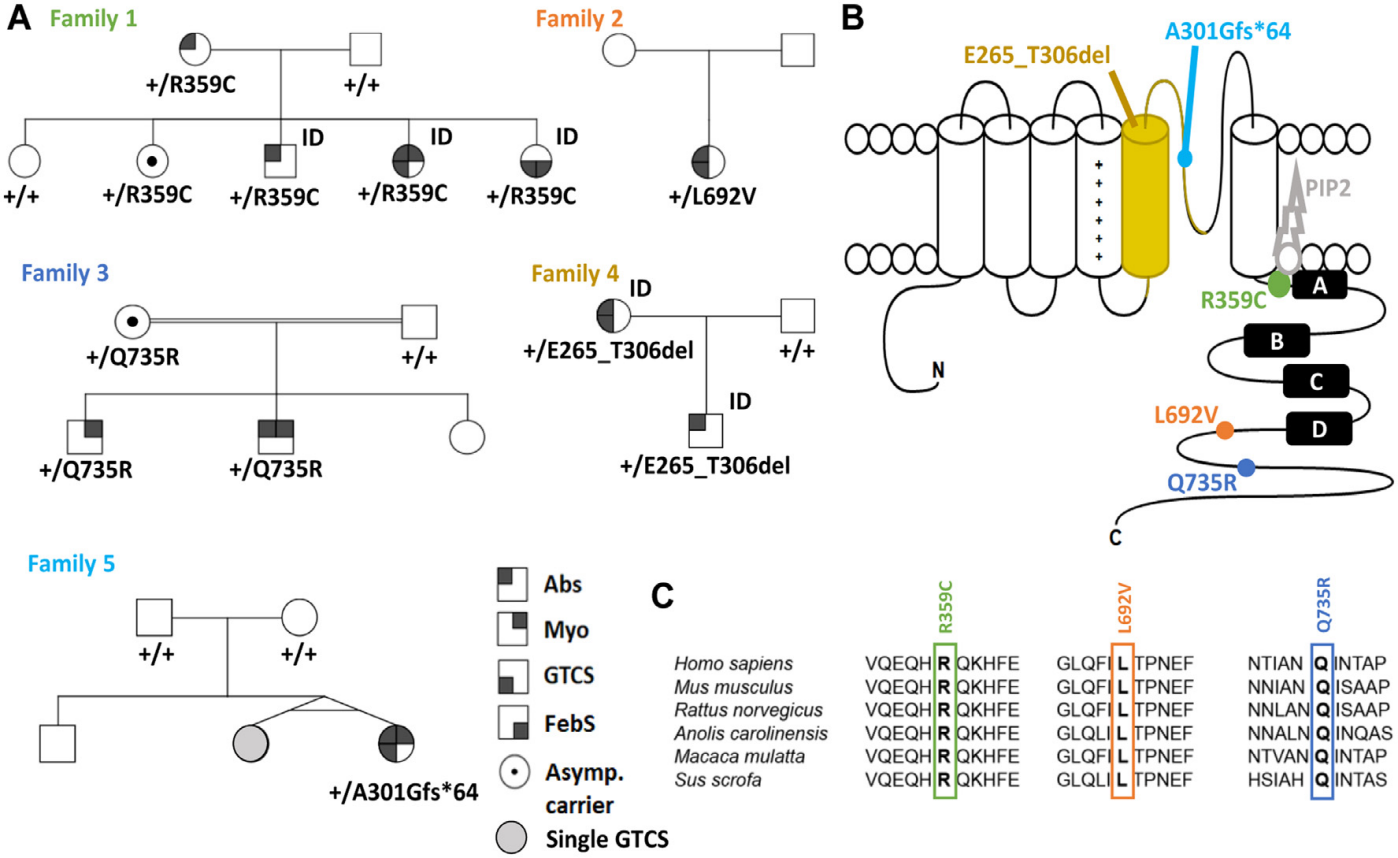
**Variants affecting the K_v_7.5 potassium channel.** (**A**) Pedigrees of patients with individuals from Table 1 indicated by number. (**B**) Schematic of the K_v_7.5 subunit of which four assemble to form a channel. Each subunit consists of a voltage-sensor domain (S1-S4) and a pore forming region (S5 and S6) including the pore loop. Point mutations are localized in highly conserved regions of the C-terminus (R359C, L692V, Q735R), while a splice site variant causes a deletion of the S5 segment and parts of the pore forming loop (E265_T306del) and a frame-shift mutation leads to an early stop codon in the pore loop (A301Gfs*64). (**C**) Amino acid alignment across multiple species (top) and across all human K_V_7 family members (bottom) shows evolutionary conservation of R359, L692, Q735 and their surrounding amino acids in K_V_7.5. Species from top to bottom: human, mouse, rat, Carolina anole, macaque, pig. ID = intellectual disability, Abs = absence seizure, Myo = myoclonic seizure, GTCS = generalized tonic clonic seizure, FebS = febrile seizure, asymp = asymptomatic.

In total, we identified 10 individuals within 5 families with a GGE phenotype carrying a disease-associated variant (see criteria in methods) in *KCNQ5.* No other variants in genes that have been associated with epilepsy before were detected. R359C and Q735R were first detected, each in 1 of 238 independent GGE families from the EuroEPINOMICS-CoGIE cohort.^23^ Both variants co-segregated in 2-4 affected family members and were found each also in one asymptomatic carrier. This prompted us to search for further variants in three other cohorts. L692V was found in 1 individual of 339 GGE families in the Epi25 subcohort from Tuebingen, E265_T306del in 2 related individuals in an epilepsy gene panel that was performed in 75 individuals in France, and A301Gfs*64 in 1 individual of 640 GGE families from the Epi4K/EPGP cohort.^22^ Two additional variants from the Epi4K/EPGP cohort, F165I and L926S, were found one time each in the gnomAD or TOPMed databases and did not show any alterations in electrophysiological recordings as compared to the WT (see Table 2). Hence, the individuals carrying these benign variants will not be considered further on. The pedigrees of families 1-5 are shown in Figure 1A.

Statistical analysis was performed to validate *KCNQ5* as a potentially predisposing gene for GGEs. The odds of the occurrence of qualifying variants (as previously defined by the Epi4K/EPGP^22^) in *KCNQ5* in GGE cases vs. controls was calculated over three large previously published cohorts (partially overlapping with the discovery cohorts mentioned above), each matched with independent controls. In total, we identified 8 qualifying variants in cases vs. 3 in controls, with a stratified odds ratio of 10·6 (confidence interval: 2·4-64·8; *p* = 0.00044). The odds were homogeneous across cohorts (Breslow-Day test; *p* = 0·9). This suggests that *KCNQ5* variants are enriched in GGE cases, considering this hypothesis-driven approach.

### Clinical descriptions

#### Family 1 (R359C)

The family of German descent includes four clinically affected individuals with variable manifestations. Whereas the mother and the son suffer from absence seizures only, one daughter exhibits myoclonic seizures and generalized tonic clonic seizures (GTCS) in addition to absences. The other affected daughter (individual 3) presents with GTCS alone. Age at epilepsy-onset varied between 3 and 7 years. All three affected children have ID. However, while individuals 2 and 3 already showed a developmental delay or ID before the onset of epilepsy, ID in individual 1 started after the onset of epileptic seizures. The mother has a normal intellect. One non-affected sibling carries the variant too, indicating reduced penetrance. Seizure control differs between the individuals of family 1 though lamotrigine seemed to have a positive effect in all affected family members. Relevant comorbidities have only been observed in the most severely affected individual (individual 1), who suffers from early onset arterial hypertension, obesity and mild generalized cerebellar ataxia.

Routine-EEG in individual 1 and 2 revealed generalized irregular 3/s (poly-)spikewave complexes, photoparoxysmal reaction and eyelid myoclonia under photostimulation. Generalized spike waves have also been present in individual 3, EEG recordings of individual 4 were not available. Cerebral imaging with MRI or CT-scan has not been performed in any of the family members. Clinical features of all our individuals are summarized in Table 1.

**Table 1:** Clinical features of individuals with disease-associated variants in *KCNQ5*.

	Individual 1	Individual 2	Individual 3	Individual 4	Individual 5	Individual 6	Individual 7	Individual 8	Individual 9	Individual 10
Family	1				2	3		4		5
**Descent**	German	German	German	German	Chechen	Turkish	Turkish	French	French	Australian-European
**Epilepsy Syndrome**	CAE->JME	CAE	IGE-GTCS	CAE	CAE->IGE-GTCS	JME	JME	CAE	CAE-GTCS	JME
**Variant**	1075C>T	1075C>T	1075C>T	1075C>T	2074C>G	2204A>G	2204A>G	918+5G>A	918+5G>A	901dupG
	R359C	R359C	R359C	R359C	L692V	Q735R	Q735R	E265_T306del	E265_T306del	A301Gfs*64
**Functional consequence**	LOF	LOF	LOF	LOF	LOF	LOF	LOF	LOF	LOF	LOF
**Consanguinity**	No	No	No	No	No	parents first degree cousins	parents first degree cousins	No	No	No
**Sex, age at investigation**	Female 19y	Male 6y	Female 14y	Female 43y	Female 27y	Male 37y	Male	Male 4y	Female 31y	Female 48y
**Age of onset**	3y	5y	7y	7y	7y	14y	16y	18m	19m	14y
**Seizure type (at onset)**	Absence, Myoclonia, GTCS	Absence	GTCS	Absence	Absence, GTCS	Myoclonus, GTCS	Myoclonus±GTCS, Absence	Absence	Absence, GTCS	Myoclonus, GTCS, Absence
**Seizure outcome (2020)**	Ongoing seizures with VPA+LTG+ETX	Seizure free with LTG	Seizure free with LTG	Seizure free without medication	Not seizure free due to incompliance (VPA+LEV)	2-3 myoclonic seizures/week with VPA+TPM	n/a	Rare absences with VPA+LEV	Seizure free with LEV	Seizure free with LEV+LTG+ZNS
**Development before seizure onset**	Normal	Mild developmental delay starting between 3,5y and 5 y	Mild ID	Normal	Normal	Normal	Normal	Mild ID	Mild ID	Normal
**Development**	Moderate ID	Mild global developmental delay	Mild ID	Normal	Normal	Normal	Normal	Mild ID	Mild ID	Normal
**Previous or active medication**	Pharmaco-resistant partial response to VPA+LTG+ETX	Received VPA 25 mg/kg, outcome unknown	Good response to VPA+LTG	n/a	VPA+LEV	Pharmaco-resistant VPA, TPM	n/a	VPA+LEV	LEV	LEV, LTG, ZNS
**Comorbidities**	Arterial hypertension, Obesity	None	Mild tremor (VPA)	None	Migraine with Aura, PNES, depression	n/a	n/a	None	Hypothyroidy	Depression
**Neurological examination**	Mild ataxia	Normal	Normal	n/a	Normal	Normal	Normal	Normal	Normal	Normal
**EEG**	GSW, PPR(12Hz) gen. irreg. 3/s poly SW for 15sec, eyelid myoclonia under phs	irreg. GSW; PPR gen. Irreg. Poly SW, eyelid myoclonia (1Hz)	GSW	n/a	GSW	GED	n/a	GSW	Normal	PSW, photoparoxysmal response (clinically photosensitive)
**MRI/CT**	n/a	n/a	n/a	n/a	MRI normal	CT normal	n/a	MRI normal	MRI normal	MRI normal

CAE, childhood absence epilepsy; JME, juvenile myoclonic epilepsy; IGE, idiopathic generalized epilepsy; GTCS, generalized tonic clonic seizure; LOF, loss-of-function; LTG, lamotrigine; ID, intellectual disability; VPA, valproic acid; ETX,ethosuximide; LEV, levetiracetam; TPM, topiramate; ZNS, Zonisamide; PNES, psychogenic non-epileptic seizures; GSW, generalized spike-waves; PPR, photoparoxysmal response; phs, photostimulation; SW, spike-wave; irreg., irregular; GED,generalized epileptiform discharges; PSW, polyspike-and-slow-wave; n/a, not available.

#### Family 2 (L692V)

Individual 5 is the only affected family member in family 2 and is of Chechen origin. She started to have absence seizures at the age of seven years, and later had GTCS. She is not seizure-free and never took anti-seizure medication (ASM) regularly, so we cannot comment on ASM response. Development was normal. She also suffered from migraine with aura. Physical examination was normal. In the EEG she presented generalized spike-waves and MRI was described as normal. Paternal genotypes could not be established as the trace to the parents has been lost during the Chechen war.

#### Family 3 (Q735R)

In this consanguineous Turkish family (parents are first-degree cousins), two affected brothers were diagnosed with juvenile myoclonic epilepsy (JME) with a typical age of onset (14 or 16 years) of myoclonic seizures, and later GTCS. One of the brothers (individual 7) also presented with absence seizures. The variant was inherited from the unaffected mother. For individual 6 generalized epileptiform discharges were recorded on EEG. Individual 7 did not undergo EEG or cerebral imaging, while a cerebral CT scan of individual 6 was normal. Diagnosis of individual 7 was based on seizure description and positive family history. Development and neurological examinations were normal in both individuals. Individual 6 is pharmaco-resistant, whereas individual 7 is not (more definitive information on ASM regimen was not available).

#### Family 4 (E265-T306del)

Both affected individuals of the French family started to have absence seizures at the age of one and a half years (18 and 19 months, respectively). The mother (individual 9) also suffered from GTCS. They share a similar phenotype since both present with mild ID and similar age of onset (see Table 1). Individual 8 still has rare absences, individual 9 is seizure-free under medication. Neurological examination and MRI scans were normal in both. The EEG of individual 8 showed generalized spike-waves, the EEG of individual 9 was normal.

#### Family 5 (A301Gfs*64)

Individual 10, Australian born and of European descent, was diagnosed with JME (myoclonic seizures, absences, GTCS) at the age of 14 years. While neurological examination, cognitive status and MRI were normal, the EEG showed polyspike-slow-wave patterns and photoparoxysmal responses. She is seizure-free under medication (see Table 1). Interestingly, her twin sister, had a single GTCS induced by flashing lights in a discotheque at the age of 19 years. The twin sister refused genetic testing.

#### Functional characterization of *KCNQ5* variants

The *KCNQ5* c.918+5G>A (E265_T306del) variant in individuals 8 and 9 affects a donor splice-site. Splicing prediction tools predicted abolition of the donor site (SpliceAI score 0·98). To examine the consequences of this variant, RNA was extracted from blood, and analysis of cDNA amplicons with primers located in exon 2 and at the junction of exon 6 and 7 was performed and revealed the expected 583 bp band for the wild type (WT), and an additional smaller band of 460 bp, corresponding to the complete skipping of exon 5. This was confirmed by sequencing of the PCR products. This band was absent from the cDNA of the controls. To this end, the variant was assumed a LOF due to the large deletion within the critical pore region of the channel (S5 segment; see Figure 1B).

**Figure 2:**
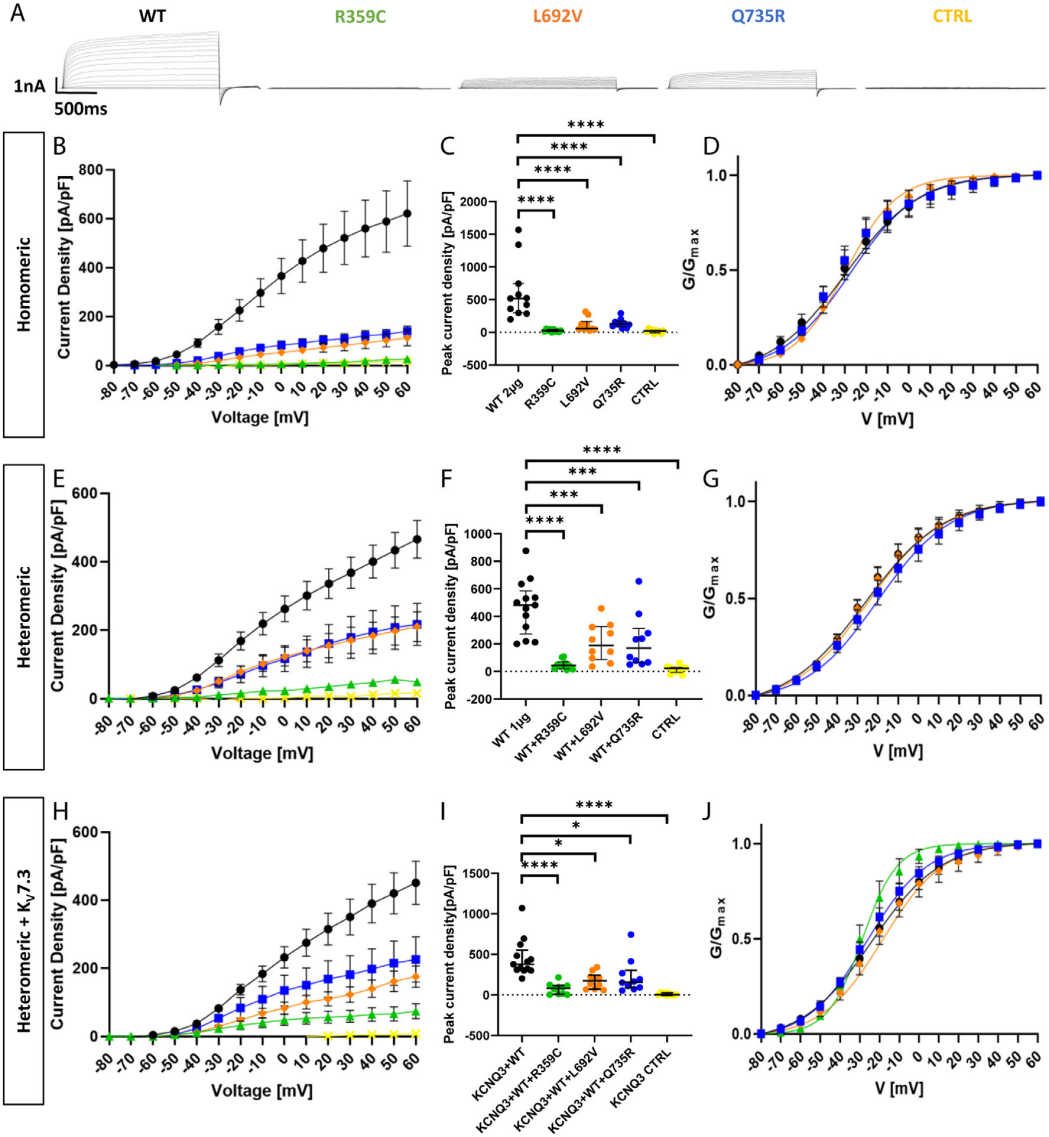
**Functional effects of K_v_7.5 WT and mutant channels in Chinese hamster ovarian cells.** (**A**) Representative K^+^ current traces from KCNQ5 WT (black), R359C (green), L692V (orange), Q735R (blue) and untransfected control cells (CTRL, yellow) during voltage steps from -80 mV to +60 mV in 10 mV increments. (**B**) Peak K+ currents of cells either transfected with WT or one mutated channel subunit were normalized by cell capacitances and plotted versus voltage. All variants result in a significant reduction in current density compared to the WT. WT, *n =* 11; R359C, *n =* 10; L692V, *n =* 10; Q735R, *n =* 10; CTRL, *n =* 10. (**C**) Comparison of maximum peak current density at +60 mV. All variants show a significant reduction compared to the WT. (**D**) Voltage-dependent activation curves. Lines represent Boltzmann functions fit to the normalized tail current. Currents in the R359C variant were too small to establish such a relationship. (**E**) Peak K+ currents normalized by cell capacitances and plotted versus voltage of cells either transfected with WT (1 µg) or WT and one mutated channel subunit (1 µg + 1 µg). The significant reduction persisted in all variants compared to the WT indicating a dominant negative effect of the variants on the WT. WT, *n =* 12; WT + R359C, *n =* 10; WT + L692V, *n =* 10; WT + Q735R, *n =* 10; CTRL, *n =* 10. (**F**) Comparison of maximum peak current density at +60 mV. All variants show a significant reduction compared to the WT. (**G**) Voltage-dependent activation curves. Lines represent Boltzmann functions fit to the normalized tail current. Currents of the WT + R359C were still too small to establish such a relationship. (**H**) Peak K+ currents normalized by cell capacitances and plotted versus voltage of cells either transfected with WT (1 µg) or WT and one mutated channel subunit (1 µg + 1 µg) in a CHO line stably transfected with KCNQ3-WT. The dominant negative effect of the variants on the WT and the significant reduction persisted in all variants compared to the WT. WT, *n =* 13; R359C, *n =* 10; L692V, *n =* 10; Q735R, *n =* 10; CTRL, *n =* 10. (**I**) Comparison of maximum peak current density at +60 mV. All variants show a significant reduction compared to the WT. (**J**) Voltage-dependent activation curves. Lines represent Boltzmann functions fit to the normalized tail current. Shown are means ± SEM (**B, D, E, G, H, J**). Scatter-and-whisker plots (**C, F, I**) show median (horizontal line) and the interquartile ranges. Dots indicate maximum values of single cells. *p ≤ 0·05; ** *p ≤* 0·01; *** *p ≤* 0·001; **** *p ≤* 0·0001; Table 2 provides exact values and statistical analyses.

To functionally characterize the remaining variants, CHO cells were transfected either with the mutant *KCNQ5* cDNA and compared to those transfected with WT *KCNQ5* cDNA (2-2·5 µg), or with a 1:1 mix of mutant and WT cDNA (1 µg each) and compared to the same amount of WT cDNA alone (1 µg) to test for a dominant-negative effect of mutant on WT channels. Standard whole-cell patch-clamp recordings were performed from transfected cells which were identified via fluorescent markers localising in the cytosol as they are cleaved off from the subunit by a P2A cleavage site to not affect channel function. Figure 2A displays representative raw current traces recorded from cells expressing either WT or one of the mutant channel subunits. Untransfected cells were used as additional controls. Homomeric expression of all three mutated channel subunits caused a significant reduction in peak current amplitude and current density (Figure 2B). The R359C variant presented the most severe reduction being almost indistinguishable from untransfected control cells (peaks at 25·9 ± 5·9 pA/pF and 15·5 ± 8·4 pA/pF, respectively; both *n = 10*; see Table 2). L692V and Q735R reached comparable current densities (112·3 ± 32·2 pA/pF and 139·6 ± 21·4 pA/pF, respectively; both *n = 10*), of about 20 % of the WT (620·9 ± 133·3 pA/pF; *n = 11*; Figure 2C). The voltage dependence of activation, as derived from normalized tail currents, was not changed for L692V and Q735R, and could not be evaluated for R359C, since the currents exhibited by channels carrying this variant were too small to be evaluated (Figure 2D and Table 2).

**Table 2:** Biophysical properties of K_v_7.5 WT and variant channels.

		Homomeric expression					Heteromeric expression					Heteromeric expression + KCNQ3-WT				
				Activation kinetics					Activation kinetics					Activation kinetics		
Subcohort		Current density [pA/pF]	*n*	V_1/2_ [mV]	k	*n*	Current density [pA/pF]	*n*	V_1/2_ [mV]	k	*n*	Current density [pA/pF]	*n*	V_1/2_ [mV]	k	*n*
- antibiotics	**WT**	620·9 ± 133·3	11	-29·91 ± 5·26	-17·31 ± 1·93	11	487·8 ± 54·6	12	-26·40 ± 3·16	-19·26 ± 3·32	12	451·1 ± 44·0	13	-24·05 ± 3·17	-17·60 ± 2·13	13
	**R359C**	25·9 ± 5·9^a^	10	-	-	-	49·1 ± 10·7^a^	10	-	-	-	106·8 ± 32·5^c^	10	-28·68 ± 3·58	-8·80 ± 0·56	7
	**L692V**	112·3 ± 32·2^a^	10	-29·51 ± 2·66	-11·98 ± 1·67	9	210·7 ± 43·5^b^	10	-24·76 ± 3·29	-18·22 ± 2·57	10	171·5 ± 31·5^d^	10	-18·45 ± 5·97	-15·43 ± 2·33	9
	**Q735R**	139·6 ± 21·4^a^	10	-27·35 ± 6·41	-15·95 ± 3·91	9	217·5 ± 61·9^b^	10	-19·98 ± 5·45	-17·75 ± 1·56	9	226·3 ± 66·7^d^	10	-26·40 ± 2·28	-15·19 ± 2·00	9
+ antibiotics	**WT**	100·9 ± 7·65	69	-22·74 ± 2·98	-20·0 ± 1·83	34	95·57 ± 12·96	40	-17·29 ± 4·13	-26·57 ± 2·93	24	-	-	-	-	-
	**901dupG**	8·35 ± 1·91^c^	16	-	-	-	115·9 ± 17·7	27	-11·73 ± 3·99	-25·33 ± 1·64	23	-	-	-	-	-
	**F165I**	153·6 ± 25·48	19	-16·25 ± 3·34	-21·02 ± 2·31	19	-	-	-	-	-	-	-	-	-	-
	**L926S**	106·1 ± 7·74	26	-18·96 ± 3·77	-20·31	23	-	-	-	-	-	-	-	-	-	-

a *p* < 0·0001 via one-way ANOVA with post hoc correction for multiple comparisons with Dunnett's test.

b *p* < 0·001 via one-way ANOVA with post hoc correction for multiple comparisons with Dunnett's test.

c *p* < 0·0001 via Kruskal-Wallis test with post hoc correction for multiple comparisons with Benjamini, Krieger and Yekutieli's test.

d *p* < 0·05 via Kruskal-Wallis test with post hoc correction for multiple comparisons with Benjamini, Krieger and Yekutieli's test.

Heteromeric expression of mutant and WT subunits in a 1:1 ratio did increase the current density for all variants compared to homomeric expression (Figure 2E). We observed a dominant negative effect for all three variants which was most severe for R359C (peak current density of 49·1 ± 10·7 pA/pF, *n = 10*) corresponding to 10 % of the WT amplitude (487·8 ± 54·6 pA/pF; *n = 12*), while L692V and Q735R reached less than 45 % of the WT (210·7 ± 43·5 pA/pF and 217·5 ± 61·9 pA/pF, respectively; both *n = 10*) (Figure 2F). The voltage dependence of channel activation was again similar for L692V/WT, Q735R/WT and WT alone, while R359C/WT-associated amplitudes were still too small for data evaluation (Figure 2G).

Since K_v_7.5 and K_v_7.3 subunits can form heterotetramers,^1^^,^^2^ heteromeric co-expression of K_V_7.5 WT and mutant subunits in a 1:1 ratio (1 µg of each clone) was performed in a CHO cell line stably expressing K_V_7.3 WT channels. Antibiotics were removed from the medium 72 hours prior to recordings to ensure comparability with the previous recordings that were conducted in cells cultured in antibiotics-free medium. Current densities were still significantly reduced for all three investigated variants indicating dominant-negative effects also under these conditions. Peaks reached 24 % of the WT (451·1 ± 44·0 pA/pF; *n = 13*) for R359C (106·8 ± 32·5 pA/pF; *n = 10*), 38 % for L692V (171·5 ± 31·5 pA/pF; *n = 10*), and 50 % for Q735R (226·3 ± 66·7 pA/pF; *n = 10*; Figure 2H and I). Activation curves of all three variants did not significantly differ from the WT (Figure 2J).

**Figure 3:**
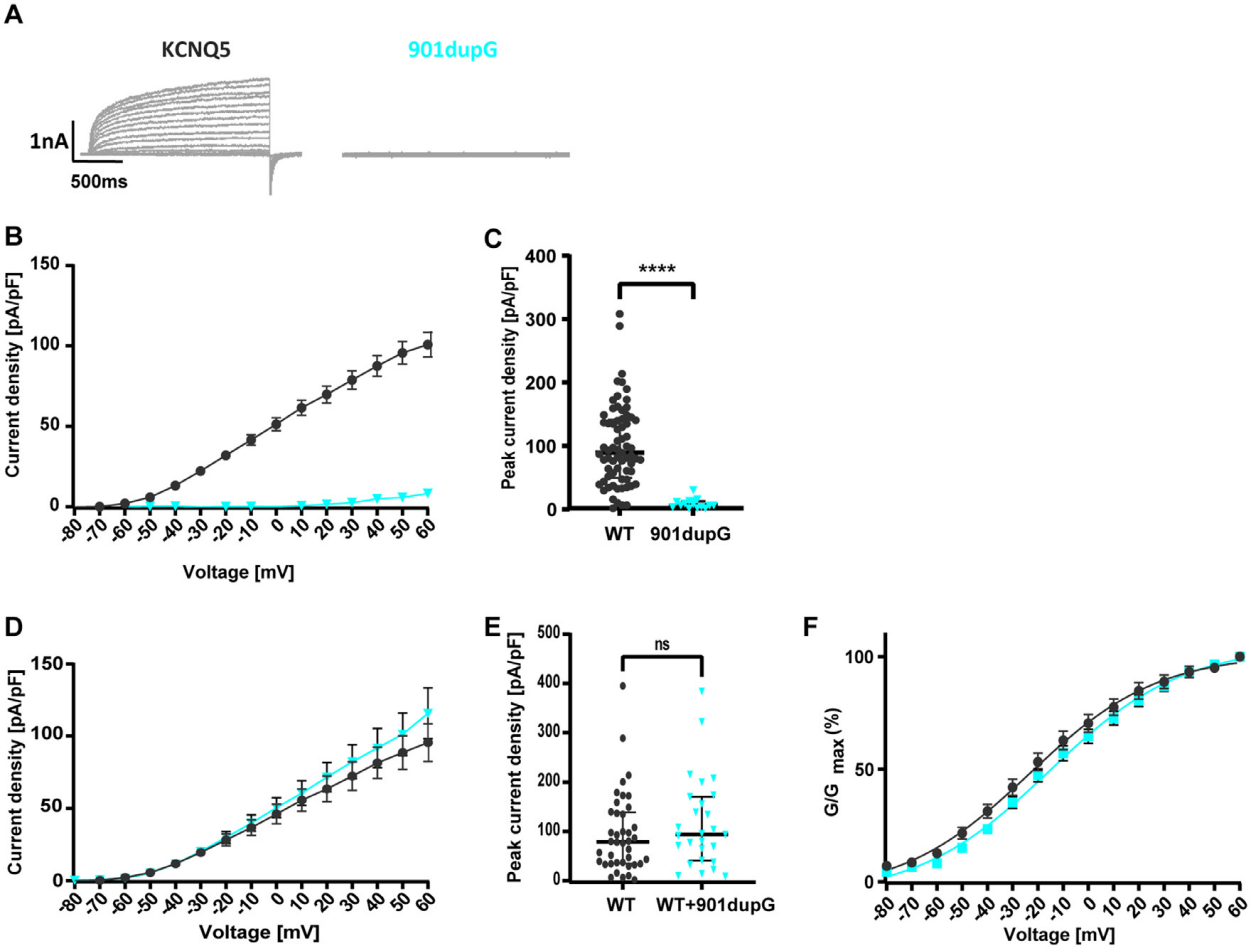
**Functional effects of K_v_7.5 WT and mutant channels in Chinese hamster ovarian cells (+ antibiotics cohort).** (**A**) Representative K^+^ current traces from KCNQ5 WT (black) and c.901dupG (turquoise) during voltage steps from -80 mV to +60 mV in 10 mV increments. (**B**) Peak K+ currents of cells either transfected with WT or c.901dupG channel subunit were normalized by cell capacitances and plotted versus voltage. WT, *n =* 70; 901dupG, *n =* 16. (**C**) Comparison of maximum peak current density at +60 mV. c.901dupG results in a significant decrease in current density compared to the WT. (**D**) Peak K+ currents normalized by cell capacitances and plotted versus voltage of cells either transfected with WT (2·5 µg) or WT and 901dupG (2·5 µg + 2·5 µg). No significant differences were observed. WT, *n =* 40; WT+901dupG, *n =* 27. (**E**) Comparison of maximum peak current density at +60 mV. No significant differences were observed. (**F**) Voltage-dependent activation curves. Lines represent Boltzmann functions fit to the normalized tail current. Shown are means ± SEM (**B, D, F**). Scatter-and-whisker plots (**C** and **E**) show median (horizontal line) and the interquartile ranges. Dots indicate maximum values of single cells. **** *p ≤* 0·001; ns non-significant; Table 2 provides exact values and statistical analyses.

In a second series of experiments, the effect of the truncating variant A301Gfs*64 (corresponding to c.901dupC) and two additional missense variants (F165I and L926S) were investigated. As these cells were cultured under different conditions using antibiotics in the culture medium (see Materials and Methods), the results of these experiments are shown separately as a second cohort (+ antibiotics), since antibiotics can decrease current density (see Table S1 and Fig S1) . Homomeric expression caused a complete LOF (current density 8·35 ± 1·91 pA/pF; *n = 16;* Figure 3A to C) compared to the WT (100·9 ± 7·65 pA/pF; *n = 69*), similar to the effect of the R359C. When co-expressed with WT subunits, neither current density nor activation curves were significantly different in cells expressing the WT subunit alone versus cells expressing WT and variant (see Figure 3D to F and Table 2). Consequently, WT and variant were not co-expressed in the cell line stably expressing K_V_7.3, due to the missing effect of the variant on the K_V_7.5 WT caused by the absence of the C-terminus due to the variant, and hence, its presumed inability to form heteromers with the WT channels. Both additional missense variants did not show a significant difference in either current density or gating parameters as compared to the WT (see Table 2).

In summary, two missense variants were found to have no functional effect and were thus considered benign, while three other missense variants cause a dominant-negative LOF effect by reducing current density in all three expression conditions, the R359C being the most severe one. The truncated variant (A301Gfs*64) has no effect on the WT subunits, only causing a haploinsufficiency. The voltage dependence of channel activation was not significantly changed for any of the variants (Table 2 ).

## Protein production and membrane expression of *KCNQ5* variants in CHO cells

**Figure 4:**
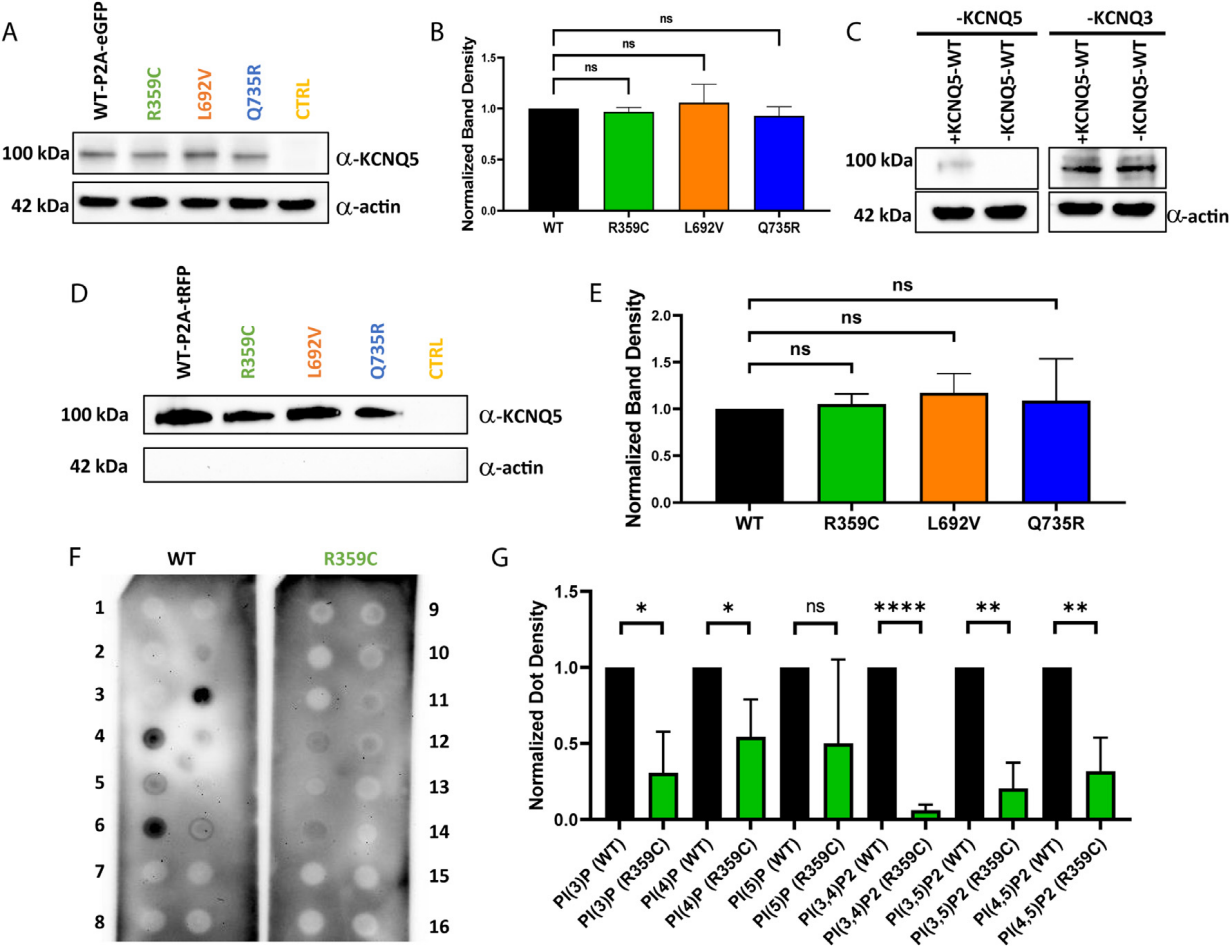
**Western blot analysis of *KCNQ5* expression and phospholipid binding abilities in CHO cells.** (**A**) Western blot of CHO cell lysates of transiently transfected cells (20µg per lane; *n =* 3). Controls consisted of untransfected CHO cells. (**B**) Comparison of K_V_7.5-WT expression to variants showed no significant changes (*n =* 3). (**C**) Western blot of K_V_7.3 and K_V_7.5 expression in the K_V_7.3 stable cell line (*n =* 4). (**D**) Biotinylation assays of transfected CHO cells for cell surface expression analysis (*n =* 3). (**E**) No significant changes in cell surface expression between K_V_7.5-WT and K_V_7.5 variants were observed (*n =* 3). Controls consisted of untransfected CHO cells. (**F**) Representative PIP strips of the phospholipid interaction of WT versus R359C (*n =* 3). 1 = Lysophosphatic acid, 2 = Lysophosphatidylcholine, 3 = Phosphatidylinositol, 4 = Phosphatidylinositol 3-phosphate, 5 = Phosphatidylinositol 4-phosphate, 6 = Phosphatidylinositol 5-phosphate, 7 = Phosphatidylethanolamine, 8 = Phosphatidylcholine, 9 = Sphingosine 1-phosphat, 10 = Phosphatidylinositol 3,4-bisphosphate, 11 = Phosphatidylinositol 3,5-bisphosphate, 12 = Phosphatidylinositol 4,5-bisphosphate, 13 = Phosphatidylinositol (3,4,5)-trisphosphate, 14 = Phosphatidic acid, 15 = Phosphatidylserine, 16 = blank. (**G**) Quantitative analysis of interactions on PIP strips (*n =* 3) using Student's unpaired t-test. ns non-significant; *p ≤ 0·05; ** *p ≤* 0·01; *** *p ≤* 0·001; **** *p ≤* 0·0001.

To investigate whether the LOF was caused by a dysfunction in channel opening, or by a trafficking or other defect, the amount of produced protein of the R359C, L692V and Q735R variants compared to the WT was determined in CHO cells via Western blot. The A301Gfs*64 variant had to be excluded from this approach due to the missing C-terminus, which carries the antibody epitope. When whole cell lysates were blotted, no significant difference in protein amount was detected as compared to the WT (Figure 4A and B; *n = 3*). In addition, expression levels of K_V_7.3 and K_V_7.5 aubunits in the stable K_V_7.3 cell line were analysed. We observed a reduction in K_V_7.5 subunit expression as compared to K_V_7.3 (Figure 4C; *n = 4*), likely explained by less cells expressing the transiently transfected K_V_7.5 than the stably expressed K_V_7.3 subunits. To further examine if the mutant subunits were integrated in the cell membrane, a cell surface protein biotinylation assay with subsequent Western blot was performed. Again, no significant difference between the WT and the variants was observed (Figure 4D and E; *n = 3*). Consequently, the variants do neither alter overall nor cell surface expression of any of the mutant subunits, which suggests that the LOF is likely caused by dysfunctional channel opening and not by a defect in protein production, folding or trafficking.

## The R359C variant alters PI(4,5)P_2_ interaction

**Figure 5:**
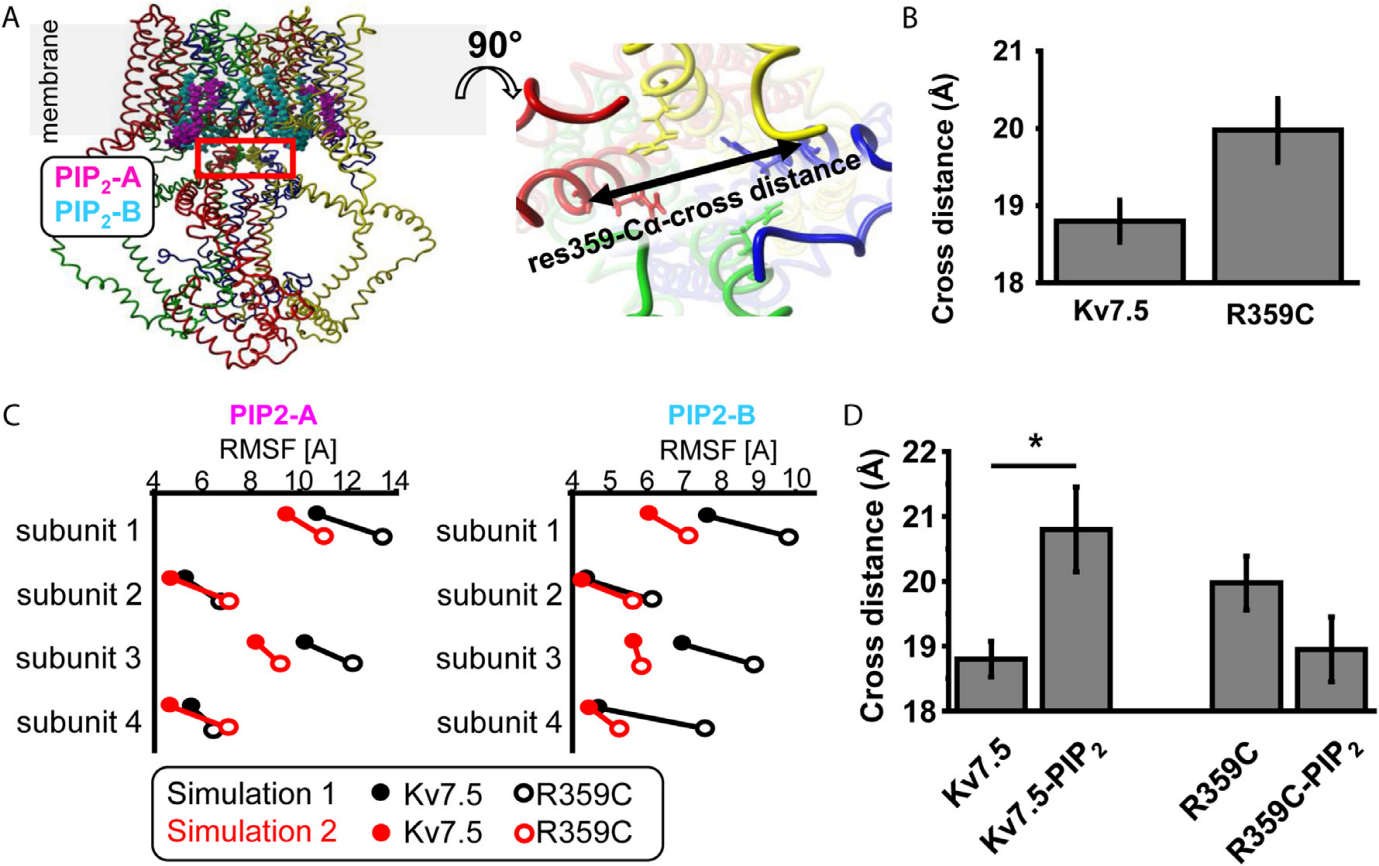
**Model predictions for K_V_7.5-WT and K_V_7.5-R359C and their interaction with PI(4,5)P_2_.** (**A**) Two K_V_7.5 consensus homology models were generated and K_V_7.5-R359C was introduced in each model. PIP_2_ molecules were positioned in the K_V_7.5-WT and K_V_7.5-R359C models in the virtually identical position as described for the K_V_7.4-WT structure 7VNP.pdb.^28^*In silico*, two PIP_2_ molecules bind to two distinct sites per K_V_7.5 subunit (PIP_2_-A is shown in magenta and PIP_2_-B is colored in cyan). The position of residues R359 are encircled by a red square (upper right), whereas each two R359 are positioned opposite in a tetrameric assembly and allow for calculation of two Cα cross distances in a membrane parallel plane (cartoon right). (**B**) Cα-cross distances in both 180° tilted directions were calculated resulting is two values per simulation. The cross distance of residue 359 tends to be slightly, however not significantly (Student's paired t-test), larger in K_V_7.5-R359C in absence of PIP_2_ in the simulations compared to the K_V_7.5-WT protein. (**C**) The more tight a PIP_2_ molecule binds the more restricted is its flexibility is which can be calculated as Root Mean Square Fluctuation (RMSF). The RMSF of PIP_2_ molecules -A and -B in the K_V_7.5-WT(filled symbols) and K_V_7.5 R359C (open symbols) channel complexes were calculated per channel subunit for both simulation approaches (simulation 1 in red and simulation 2 in black). In all simulations, both PIP_2_ binding sites for K_V_7.5-R359C mutant channels showed lower RMSF values compared to K_V_7.5-WT channels *in silico* (Paired Student's t-test, p= 0.0006). (**D**) The cross distance of K_V_7.5-R359 is significantly (Student's paired t-test) larger in presence of PIP_2_ (p=0.041) whereas this effect is not detected in the K_V_7.5-R359C.

R359 is homologous to R360 in K_v_7.1, which has been previously described as one of the key PI(4,5)P_2_ interaction sites.^30^^,^^31^ We performed homology modelling to investigate whether the dominant-negative LOF of the R359C variant in current density could be caused by a change in the interaction of K_V_7.5 channels with PI(4,5)P_2_. Homology models of K_V_7.5 and K_V_7.5-R359C in absence and presence of PIP_2_ were generated based on high homology to K_V_7.4. Two all-atoms-mobile MD simulations were conducted on each individual K_V_7.5 and K_V_7.5-R359C models (Figure 5A). The mean distance of the lower S6-helices at residue 359 tends to be increased in K_V_7.5-R359C compared to K_V_7.5 simulations due to a mild rotation (Figure 5B). As PIP_2_ is known to impact positioning of the lower S6 the simulations were repeated in the presence of PIP_2_ in position virtually identical as in K_V_7.4.^28^ Two PIP_2_ molecules (PIP_2_-A and PIP_2_-B) bind to each channel subunit. The more tight a PIP_2_ molecule binds to its binding site the less flexible it becomes. Flexibility *in silico* is calculated by fluctuations of atoms around the mean position during an MD simulation (calculated as Root Mean Square Fluctuation abbreviated as RMSF). All RMSF values of PIP_2_ molecules at each subunit in two independent simulations were decreased in the K_V_7.5-R359C model suggesting altered PIP_2_ interaction *in silico* (Figure 5C). Finally, the mean cross distance at residue 359 Cα in K_V_7.5 is increased whereas this effect is not present in K_V_7.5-R359C *in silico* (Figure 5D). Thus, mutation R359C seems to impair PIP_2_ modulation in K_V_7.5 in the lower S6 activation gate *in silico*.

Furthermore, protein-phospholipid overlay assays were performed to investigate whether we could identify altered PIP_2_ interactions *in vitro* as well. The assays showed a significant reduction in the binding affinity of R359C channels compared to the WT to phosphatidylinositol 3-phosphate (PI(3)P; *p* = 0·0113), phosphatidylinositol 4-phosphate (PI(4)P; *p* = 0·0327), and most importantly, phosphatidylinositol 3,4-bisphosphate (PI(3,4)P_2_; *p* < 0·0001 ), phosphatidylinositol 4,5-bisphosphate (PI(4,5)P_2_; *p* = 0·0060), and phosphatidylinositol 3,5-bisphosphate (PI(3,5)P_2_; *p* = 0·0012; Figure 4F and G; *n = 3*). Our results suggest that R359 plays an important role to confer binding to PI(4,5)P_2_ that is essential for channel opening, as Thomas et al. (2011) found for the homologous site in *KCNQ1*.

**Figure 6:**
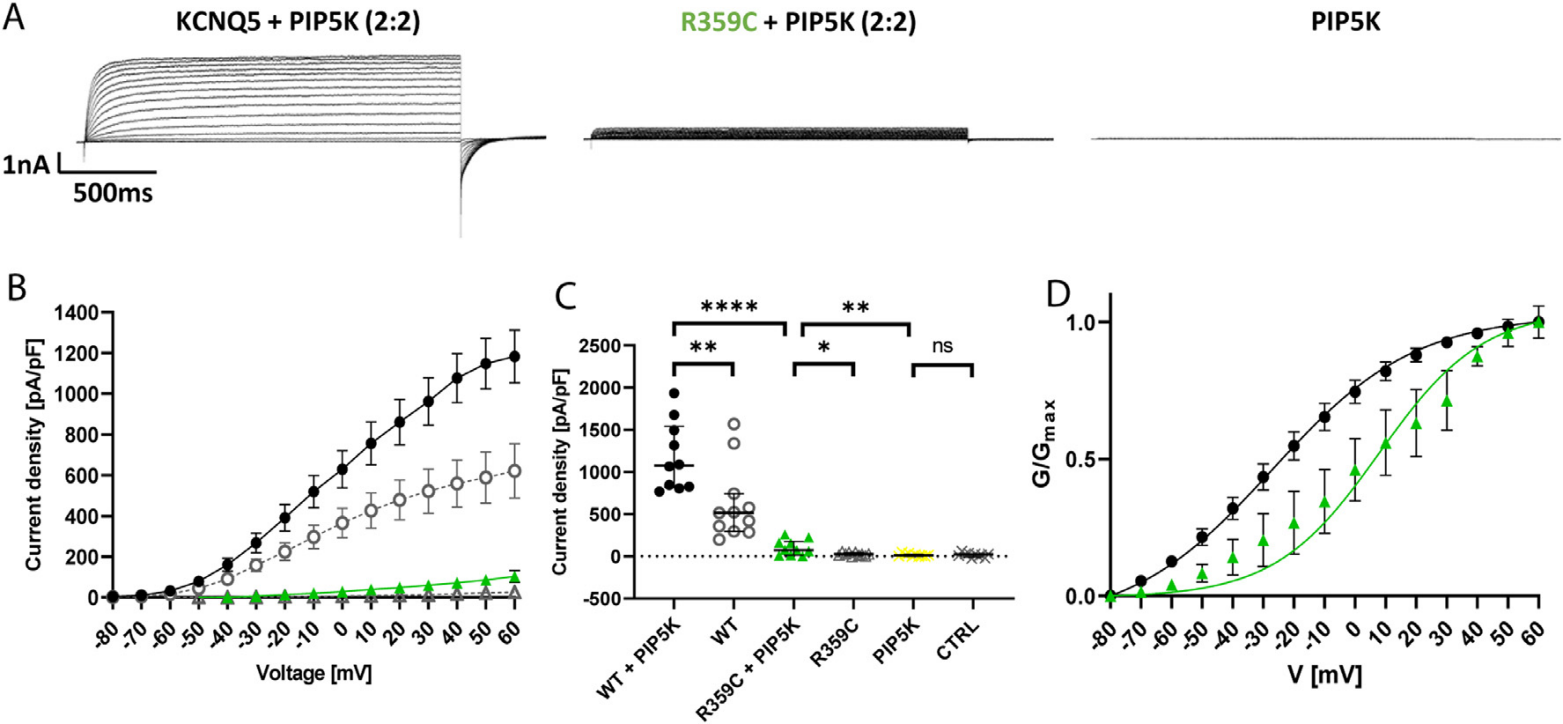
**Functional effects of PIP_2_ overexpression in K_V_7.5 WT vs. R359C channels in CHO cells.** (**A**) Representative K^+^ current traces from K_V_7.5-WT + PIP5K (black), K_V_7.5-R359C + PIP5K (green), and PIP5K control cells (black) during voltage steps from -80 mV to +60 mV in 10 mV increments. (**B**) Peak K^+^ currents of cells either transfected with WT alone (grey dots), WT + PIP5K (black), R359C (grey triangles) or R359C + PIP5K (green triangle) channel subunit were normalized by cell capacitances and plotted versus voltage. The currents generated by K_V_7.5-R359C + PIP5K remain largely reduced as compared to K_V_7.5-WT + PIP5K. K_V_7.5-WT, *n =* 12; K_V_7.5-WT + PIP5K, *n =* 10; K_V_7.5-R359C, *n =* 10; K_V_7.5-R359C + PIP5K, *n =* 10; PIP5K, *n =* 10; CTRL *n* = 10. (**C**) Comparison of maximum peak current density at +60 mV. PIP5K co-expression significantly increased the K_V_7.5-R359C peak current density, yet it is still significantly reduced as compared to K_V_7.5-WT + PIP5K. (**D**) Voltage-dependent activation curves. Lines represent Boltzmann functions fit to the normalized tail current. The activation curve for K_V_7.5-R359C + PIP5K is significantly shifted towards more positive voltages. Shown are means ± SEM (**B, D**). Scatter-and-whisker plots (**C**) show median (horizontal line) and the interquartile ranges. Dots indicate maximum values of single cells. **** *p ≤* 0·001; **p ≤ 0·01; * *p ≤* 0·05; ns non-significant; Table 3 provides exact values and statistical analyses.

To elaborate these findings, additional electrophysiological experiments were conducted. To this end, endogenous PIP_2_ levels were increased by co-expression of a 1γ type PI(4)P5-kinase as reported previously.^32^ As a result, WT amplitude and current density significantly increased (to 1182·24 ± 129·13 pA/pF; *n = 10*; see Figure 6A, B, C) as previously reported. Interestingly, the homomeric R359C channels firstly displayed measurable amplitudes under PIP5K co-expression (in 6/10 cells), however, peak current density was still significantly decreased (103·13 ± 28·96 pA/pF; *n = 10*) as compared to the WT, only reaching 8·7 % of WT levels. Furthermore, the activation curve of R359C channels was significantly shifted towards more depolarized potentials with a V_1/2_ of 7·82 ± 11·72 mV (*n = 6;* Figure 6D) as compared to WT channels (-27·19 ± 5·85 mV; *n = 10*), whereas slopes did not appear to be significantly different (see Table 3). Co-expression of PIP5K did not change activation curve parameters in WT cells as compared to homomeric expression alone. Cells only expressing PIP5K did not show measurable amplitudes and their peak current density (14·60 ± 5·35 pA/pF; *n = 10*) did not significantly differ from untransfected CHO cells. These results support the hypothesis that the R359 site might be important for PIP_2_ interaction and thus channel opening.

**Table 3:** Biophysical properties of K_v_7.5 WT and R359C channels under PIP5K.

	Homomeric expression					PIP5K co-expression				
			Activation kinetics					Activation kinetics		
	Current density [pA/pF]	*n*	V_1/2_ [mV]	k	*n*	Current density [pA/pF]	*n*	V_1/2_ [mV]	k	*n*
WT	620·9 ± 133·3	11	−29·91 ± 5·26	−17·31 ± 1·93	11	1182·24 ± 129·13#	10	−27·19 ± 5·85	−23·34 ± 2·19	10
**R359C**	25·9 ± 5·9 a	10	-	-	-	103·13 ± 28·96^a^^,^##	10	7·82 ± 11·72 #	−17·03 ± 5·50	6

a *p* < 0·0001 via one-way ANOVA with post hoc correction for multiple comparisons with Dunnett's test.

# *p* < 0·01 via unpaired t-test.

## *p* < 0·05 via unpaired t-test.

**Figure 7:**
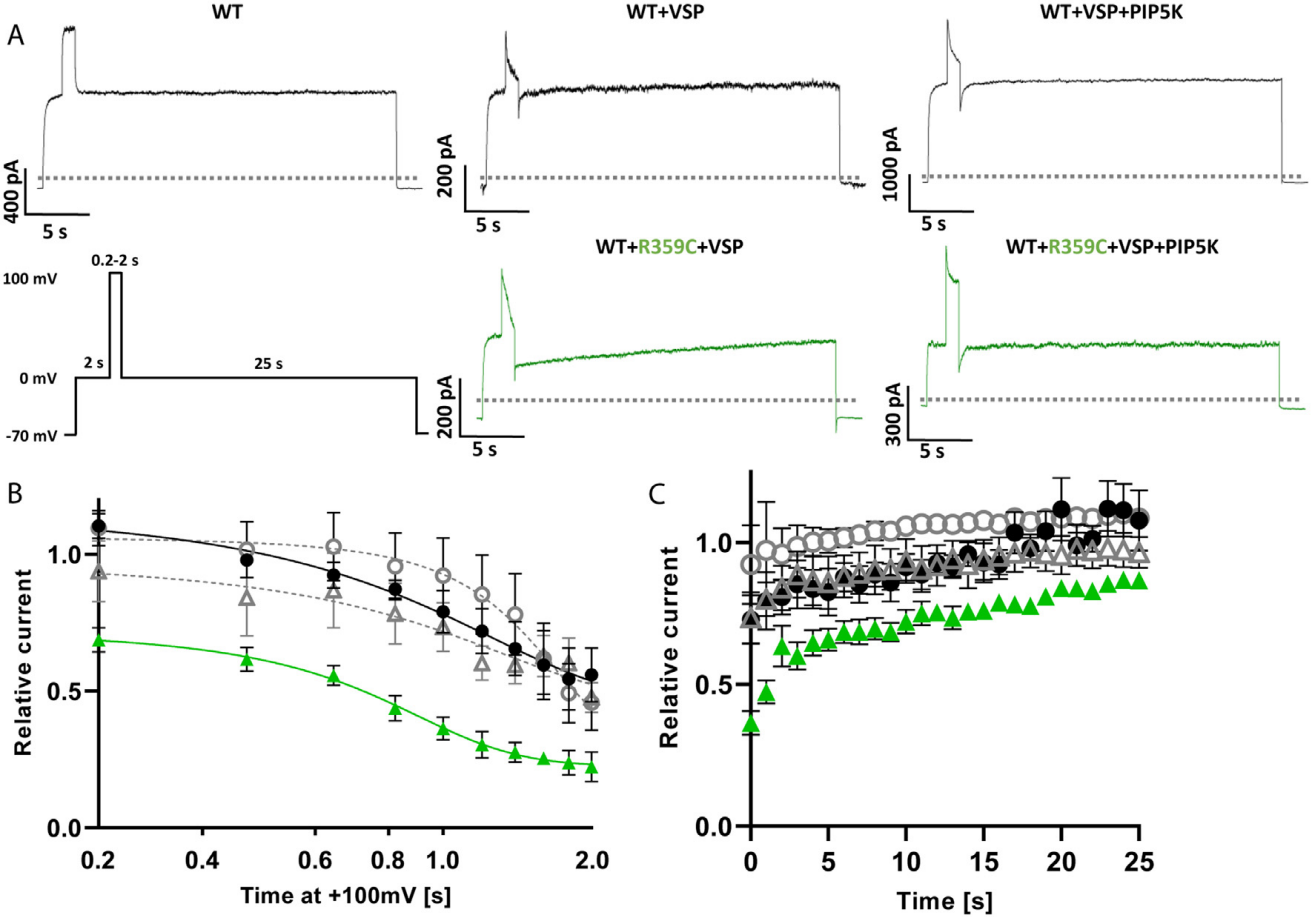
**Functional effects of PIP_2_ depletion in K_V_7.5-WT vs. K_V_7.5-R359C channels in CHO cells.** (**A**) Representative K^+^ current traces from WT (control), K_V_7.5-WT + VSP, K_V_7.5-WT + VSP + PIP5K (all black), K_V_7.5-WT + R359C + VSP, and K_V_7.5-WT + R359C + VSP + PIP5K (both green) cells responding to the displayed voltage protocol. The dotted line indicates 0 pA. (**B**) Time dependence of current decrease under VSP activation in the absence (WT black dots, R359C green triangles; *n =* 5 for both) or presence of PIP5K (WT grey dots, R359C grey triangles; *n =* 5). These values were calculated by normalizing the values immediately after the +100 mV step to those immediately prior to it and a Boltzmann function was fit to the data points. (**C**) Time dependence of current recovery after VSP activation in the absence (WT black dots, R359C green triangles; *n =* 5 for both) or presence of PIP5K (WT grey dots, R359C grey triangles; *n =* 5). These values were calculated by normalizing the values every second after the +100 mV step to that immediately prior to it. Shown are means ± SEM (**B, C**).

To further clarify a potentially decreased PIP_2_ sensitivity of this variant, additional electrophysiological recordings were conducted by co-expressing a *Daino rerio* voltage-sensing phosphatase (Dr-VSP) as described by Hossain et al. (2008), which is activated by strong depolarizations (≥ +100 mV) and temporarily reduces PIP_2_ levels in the cell membrane resulting in a temporary inhibition of K_V_7 channels.^33^ As the R359C variant suppresses currents even under WT co-expression almost completely, the stable K_V_7.3-celline was used for these experiments, in which the R359C variant yielded measurable current amplitudes in heterozygous conditions co-expressed with K_V_7.5-WT, to be able to see a potential current inhibition by VSP. Activation of VSP for only 0.2 s had an immediate effect on cells transfected with K_V_7.5-WT+K_V_7.5-R359C+VSP reducing currents by 31 %, whereas cells transfected with K_V_7.5-WT +VSP showed an effect only after 0.6 s of VSP activation (*n = 5* for all conditions; see Figure 7B) at which the current of 3/5 cells transfected with the variant does not recover from VSP activation anymore. This is further displayed in the current recovery time after VSP switch-off. While K_V_7.5-WT +VSP cells only take 3·2 ± 1·5 s to recover after 0.6 s of VSP activation and 11·8 ± 4·53 s after 1·0 s (see Figure 7A, C), K_V_7.5-WT+K_V_7.5-R359C+VSP expressing cells are significantly slowed down (19·0 ± 4·29 s; *p* < 0·01) and none of the cells recovered to the baseline value, respectively. Co-expression of PIP5K led to a quicker recovery in K_V_7.5-WT +VSP cells (1·4 ± 0·75 s and 2·6 ± 1·66 s, respectively) and was able to enhance K_V_7.5-WT+K_V_7.5-R359C+VSP cells back to normal values (6·4 ± 3·91 s and 13·4 ± 5·33 s, respectively). Consequently, the data strongly suggest that a reduction in PIP_2_ binding affinity causes the LOF in the R359C variant.

## Discussion

Here we analysed clinical and genetic data from multiple cohorts of altogether 1,292 independent families with GGE to identify and functionally characterize five likely disease-related variants suggesting that either haploinsufficiency or dominant-negative effects of *KCNQ5* are associated with GGE with or without mild to moderate ID. A hypothesis-driven statistical evaluation in three large GGE cohorts and matched controls indicated that rare, functionally relevant variants in *KCNQ5* might be more frequent in GGE than expected by chance. Since *KCNQ5* was not among the top-ranking genes in the exome-wide primary analyses of these studies,^22^^,^^23^^,^^25^ it appears that pathogenic variants in *KCNQ5* are rare.

The phenotypes ranged from mild CAE to pharmaco-resistant early-onset absence epilepsy or JME with moderate ID. Most of the individuals (8/10) presented with absence seizures. In four individuals, developmental delay or ID was present prior to epilepsy. The phenotype comprising absence seizures and ID is in accordance with a previous report of an individual with mild ID and absence seizures in adolescence, carrying an intragenic duplication in *KCNQ5* leading to haploinsufficiency.^19^ Lehman and colleagues reported four individuals suffering from ID and/or epilepsy caused by *KCNQ5* variants, which they described as LOF and one GOF (P369R) carried by the most severely affected individual. However, a recent study showed that these variants along with four additional missense variants found in children with mild to severe ID and epilepsy in fact caused a GOF in channel activation and deactivation. Two additional truncating variants including a LOF were found in individuals with a milder phenotype. Moreover, this study shows that the more severe GOF variants were found in more severely affected individuals, while milder GOF variants and the truncating LOF variants belonged to individuals with a milder phenotype.^17^ This is in line with a second recent publication that found two pore variants to cause a GOF in individuals with DEE.^18^ The individuals presented here show a milder phenotype as compared to the three studies mentioned and two carriers were even asymptomatic (one with R359C and one with Q735R). While global developmental delay of varying degree is a common feature in all descripted individuals in the above mentioned studies, three of our variants were not associated with ID (L692V, Q735R and A301Gfs*64) and the family carrying the R359C variant shows a spectrum with one individual being unaffected to individuals with mild to moderate ID. Interestingly, all four individuals from Lehman et al. suffered from ataxia with a varying degree of severity.^16^ In contrast, none of the individuals described by Wei et al. and Nappi et al. had ataxia and only one individual of our cohort, the most severely affected one, had mild ataxia on neurological examination, the others did not have neurological abnormalities on examination. Moreover, all of the individuals presented here presented with generalized seizures, yet not all of the individuals described in the other studies suffer from seizures. Five of ten of the individuals presented here became seizure-free with or without medication and for one of ten individuals we did not obtain information about pharmaco-response. Lamotrigine seemed to be effective in most carriers of R359C, and one of the carriers of this variant became seizure-free without medication. Valproate, topiramate, levetiracetam, ethosuximide and zonisamide were also prescribed but there is no indication that one of the drugs is more effective than the others.

The results of our functional analysis show a LOF in current density for homomeric channels for all investigated variants, and three of the investigated variants have a dominant-negative effect on current density in heteromeric expression experiments with a near complete loss-of-function for the R359C variant. The frameshift variant (A301Gfs*64) results in a premature stop codon in the pore region deleting the entire C-terminus. As the C-terminus comprises the interaction sites for the subunits to form a channel,^34^ its absence causes this variant to be unable to form channels under homomeric expression and abolishes interaction with the WT subunits under heteromeric conditions causing a haploinsufficiency, but not a dominant-negative effect on the WT subunits. According to our electrophysiological studies, members of family 1 carried the most severe variant (R359C), leading to a severe LOF with a dominant-negative effect. We could not find clear genotype-phenotype correlations within our study, since (i) family 1 carrying the most severe variant displays a large phenotypic heterogeneity ranging from mildly to severely affected, pharmaco-resistant individuals, (ii) individuals in family 3 exhibited a similarly severe epileptic phenotype albeit the Q735R variant showed a less prominent electrophysiological dysfunction, and (iii) also the phenotype of previously reported individuals carrying variants which only caused haploinsufficiency were reported with similar or more severe phenotypes.^16^^,^^17^^,^^18^^,^^19^ This is in contrast to other K_v_7 channels, in which functionally more severe variants with dominant-negative effects cause more severe epileptic phenotypes.^35^ Looking at our results in the context of the newly published variants, it seems that individuals carrying LOF variants display milder phenotypes, while GOF variants cause more severe phenotypes, such as DEE. Additionally, the functional severity of the GOF seems to correlate with the severity in the phenotype of the patient.^17^ Similar patterns of GOF variants inducing much more severe phenotypes such as DEE and LOF variants inducing milder phenotypes such as GGE have been described for other genes such as *SCN8A,*^36^ *SCN2A*^37^ and *KCNA2.*^38^ For *KCNQ5*, it seems that any significant LOF can contribute to an epileptic phenotype or ID of varying severity, which might be influenced by other individually differing factors, such as compensatory effects, the genetic background or environmental determinants. Larger cohorts are needed to further investigate this issue.

Remarkably, all missense variants showed stable total and membrane-expressed protein levels in CHO cells as compared to the WT. The LOF in current density is thus not caused by defects prior to membrane insertion of the channel, such as an abolished tetramerization or trafficking defect, as have been described for variants in *KCNQ2* and *KCNQ3*.^35^^,^^39^ Rather, the three investigated missense variants have functional effects on channel gating, and thus, might mark important sites involved in channel opening. K_V_7 channels form the molecular basis of M-currents and are classically negatively regulated by muscarinic acetylcholine receptors via a PI(4,5)P_2_-dependent mechanism. PI(4,5)P_2_ is required for the stabilization of the open state relative to the closed state and PI(4,5)P_2_-depletion upon activation of muscarinic acetylcholine receptors leads to channel closure. Here, we provide modelling, electrophysiological and biochemical evidence that the R359C variant causes altered PI(4,5)P_2_ binding, which possibly explains the complete, dominant-negative LOF caused by this variant.

Both other variants (L692V and Q735R) that we investigated are located in an evolutionary highly conserved region of the K_v_7.5 C-terminus, which does not show any variants in unaffected individuals in multiple databases and is absent or not conserved in other K_v_7 channels, underlining the importance of this region for proper channel function in K_v_7.5. As the functional aspects of this region on channel behaviour have not been described previously, these two variants might be able to elucidate binding partners and disclose the function of the distal C-terminus in channel opening. Investigating these molecular mechanisms may open doors for new treatment options in the future, especially for pharmaco-resistant patients.

In summary, we have identified rare loss-of-function variants in *KCNQ5* in five independent families, which are likely contributing to the pathophysiology of GGE. Two variants cause haploinsufficiency, three showed a dominant-negative effect on WT K_V_7.5 and K_V_7.3 channels. We were also able to identify the importance of R359 as crucial for PI(4,5)P_2_ interaction and channel opening. Consequently, the M-current in these individuals is likely reduced causing a decrease in action potential threshold and increased excitability of neurons expressing K_V_7.5 channels, thus leading to an elevated seizure susceptibility. The types of neurons and networks that are involved need to be determined in further studies. Furthermore, identifying these LOF variants in these patients opens doors to targeted treatment using K_V_7 channel openers such as retigabine, and further studies should be conducted to investigate their effect on the variants.

## Contributors

JKr, JS, PM, GL, SM and HL designed the study and experiments. JKr, JS, AL, JH, MM, GS, PY, MK, MSH, PM performed experiments and analyzed data. JKr, JS, AL, JH, MM, GS, PY, MK, SP, RK, PM, GL, SM, and HL, interpreted data. JKe, KA-K, HC, BJS, YGW, PK-K, SFB, GL, and HL recruited and phenotyped patients. JKr, JKe, GS, MK, PM, GL, SM, and HL wrote the manuscript. All authors read, revised and approved the manuscript.

## Data sharing statement

The exome sequencing data/analyses presented here are based on the use of study data from the Epi25 Collaborative (http://epi-25.org/), available with controlled access through dbGaP (https://ncbi.nlm.nih.gov/gap/), the EuroEPINOMICS-CoGIE project, and the Epi4K project.

## Declaration of interests

J. Krüger was financed by a grant from the Deutsche Forschungsgemeinschaft/German Research Foundation (DFG), during the conduct of the study; Dr. Schubert has nothing to disclose; Dr. Kegele has nothing to disclose; A. Labalme has nothing to disclose; Dr. Mao has nothing to disclose; J. Heighway has nothing to disclose; Dr. Seebohm has nothing to disclose; Dr. Yan has nothing to disclose; M. Koko reports grants from DAAD, outside the submitted work; Dr. Aslan has nothing to disclose; Dr. Caglayan has nothing to disclose; Dr. Steinhoff has nothing to disclose; Dr. Weber has nothing to disclose; Dr. Keo Kosal has nothing to disclose; Dr. Berkovic reports grants from NHMRC, during the conduct of the study; grants from UCB Pharma, grants from Eisai, grants from SciGen, personal fees from Bionomics, personal fees from Athena Diagnostics, outside the submitted work; In addition, Dr. Berkovic has a patent Methods of treatment, and diagnosis of epilepsy by detecting mutations in the SCN1A gene with royalties paid to Patent held by Bionomics Inc. Licensed to Athena Diagnostics; Genetics Technologies Ltd, a patent Diagnostic and Therapeutic Methods for EFMR (Epilepsy and Mental Retardation Limited to Females) with royalties paid to Licensed to Athena Diagnostics, and a patent A gene and mutations thereof associated with seizure and movement disorders (PRRT2) with royalties paid to Licensed to Athena Diagnostics; Dr. Hildebrand has nothing to disclose; Dr. Petrou reports personal fees and other from Praxis Precision Medicines, outside the submitted work; and Dr. Petrou works for a company, Praxis Precision Medicines that develop therapies for neurogenetic disorders such as KCNQ5 (but this is not currently under any consideration); Drs. Krause and May has report grants from the Fond Nationale de la Recherche in Luxembourg; Dr. Lesca has nothing to disclose; Dr. Maljevic has nothing to disclose; Dr. Lerche reports grants from the German Research Foundation (DFG), from the Federal Ministry for Education and Research (BMBF), grants from Foundation no epilep, during the conduct of the study; outside the submitted work, Dr. Lerche reports a grant from the Else-Kröner Fresenius Foundation (EKFS), a grant and personal fees from Bial, a grant from Boehringer Ingelheim, personal fees from Eisai, personal fees from UCB/Zogenix, personal fees from Arvelle/Angelini Pharma, personal fees from Desitin, and personal fees from IntraBio.

## Appendix Supplementary materials

Image, application 1

Image, application 2

Image, application 3
