# Supplementary material for: Loss-of-function variants in the KCNQ5 gene are implicated in genetic generalized epilepsies
Source: eBioMedicine. 2022 Sep 9;84:104244. doi: 10.1016/j.ebiom.2022.104244 (PMC9471468; doi:10.1016/j.ebiom.2022.104244)
Supplement: Supplementary file 1 [file mmc1.docx]

**Full Western blot images**

**Fig. 4A** – Full image of the Western blot of whole cell lysates of KCNQ5-transfected CHOs (Fig 4A). First photo of the gel showing the marker. Second Western blot. Third picture shows a merge of both.


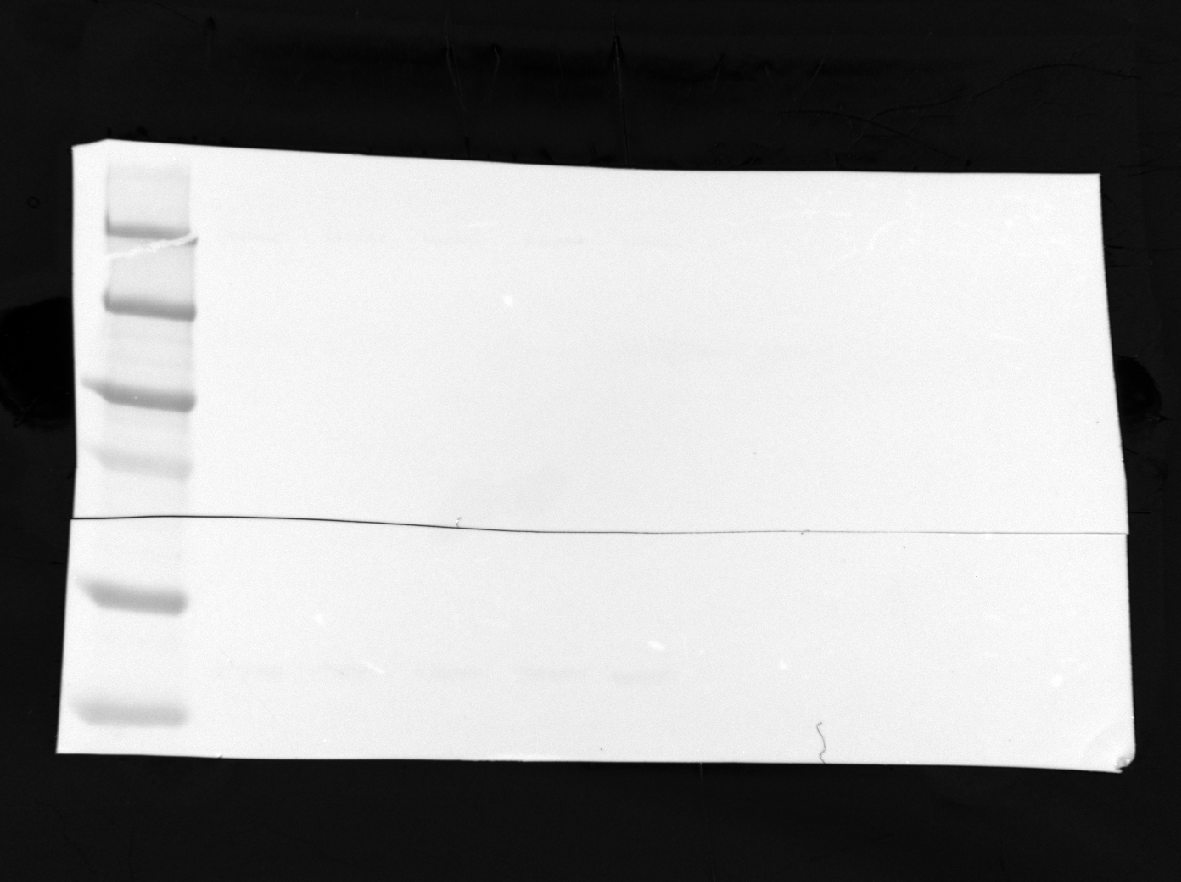

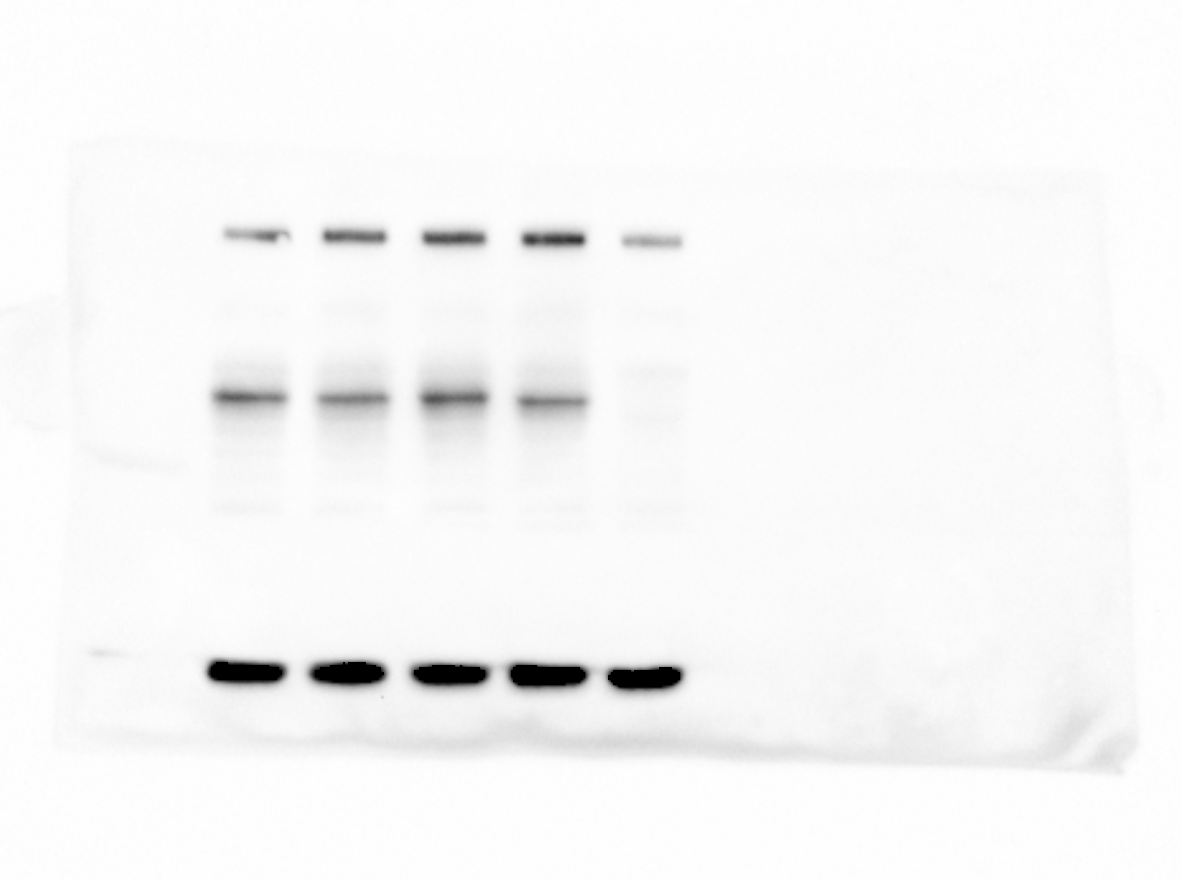

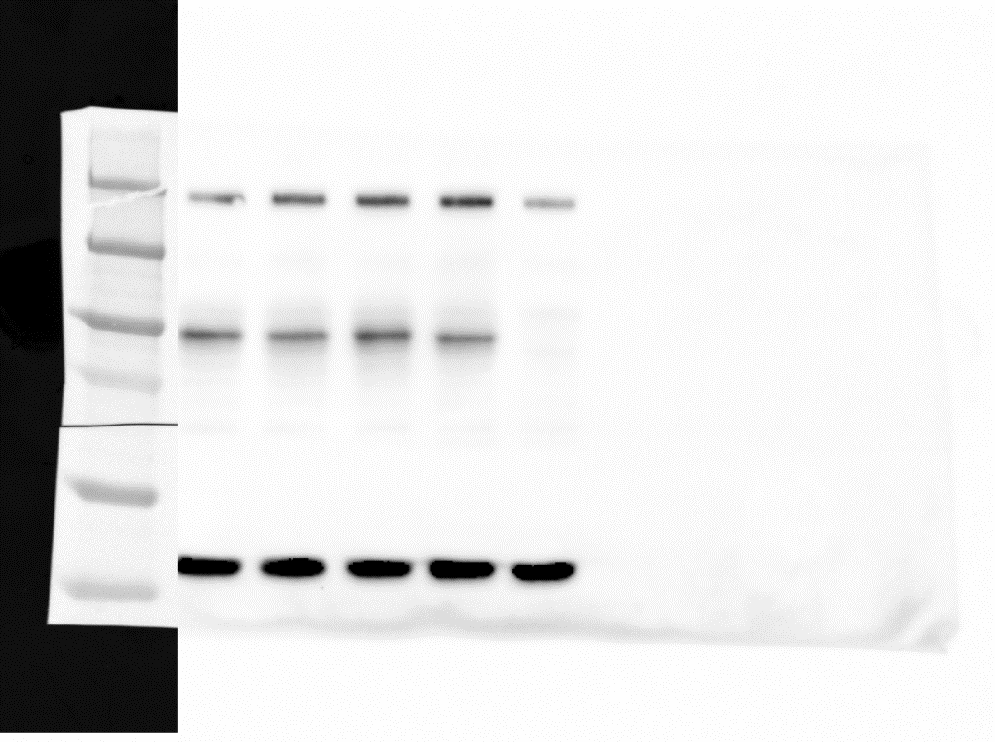


α-KCNQ5

α-actin

250 kDa

150 kDa

100 kDa

75 kDa

50 kDa

37 kDa

WT-P2A-eGFP

R359C-P2A-eGFP

L692V-P2A-eGFP

Q735R-P2A-eGFP

CTRL

**Fig. 4C** – Full image of the Western blot of whole cell lysates comparing KCNQ3 and KCNQ5 levels (Fig 4C). First photo of the gel showing the marker. Second Western blot. Third picture shows a merge of both.

37 kDa


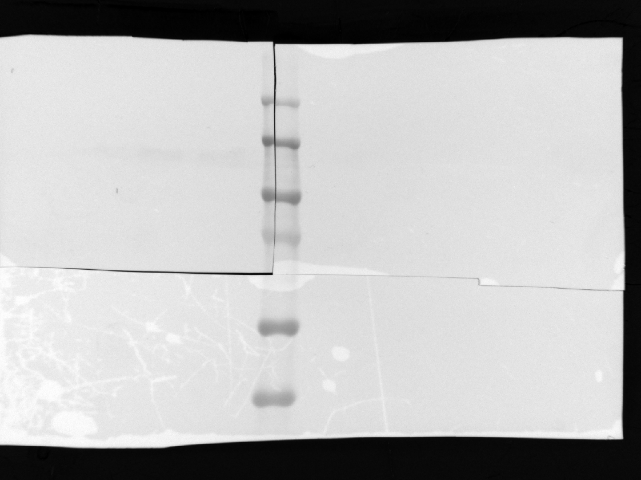

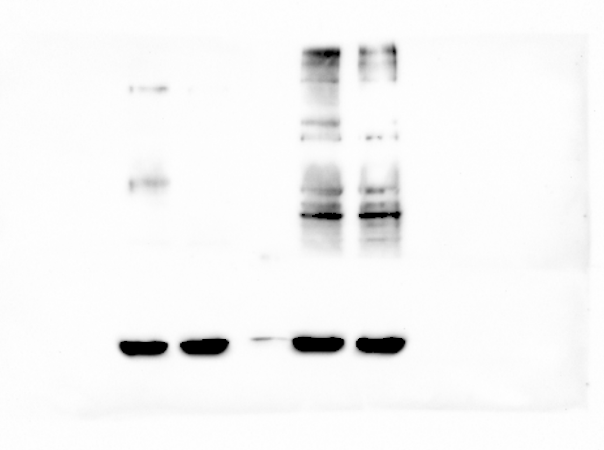


250 kDa

150 kDa

100 kDa

75 kDa

50 kDa

+KCNQ5-WT

-KCNQ5-WT

-KCNQ5-WT

+KCNQ5-WT

**
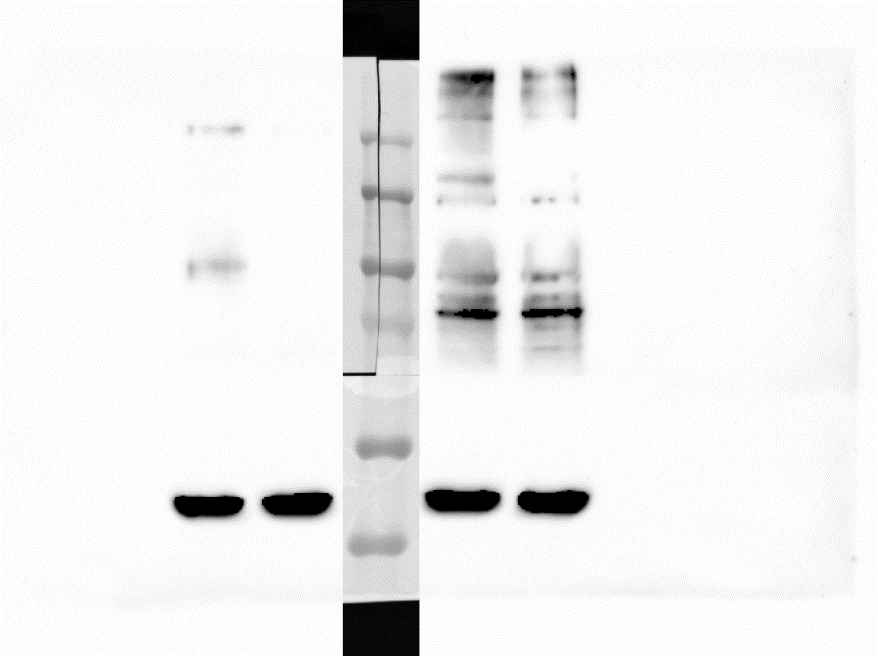
**

α-KCNQ3

α-KCNQ5

α-actin

α-actin

**Fig. 4E** – Full image of the Western blot of membrane proteins (biotinylation assays; Fig 4E). First photo of the gel showing the marker. Second Western blot. Third picture shows a merge of both.


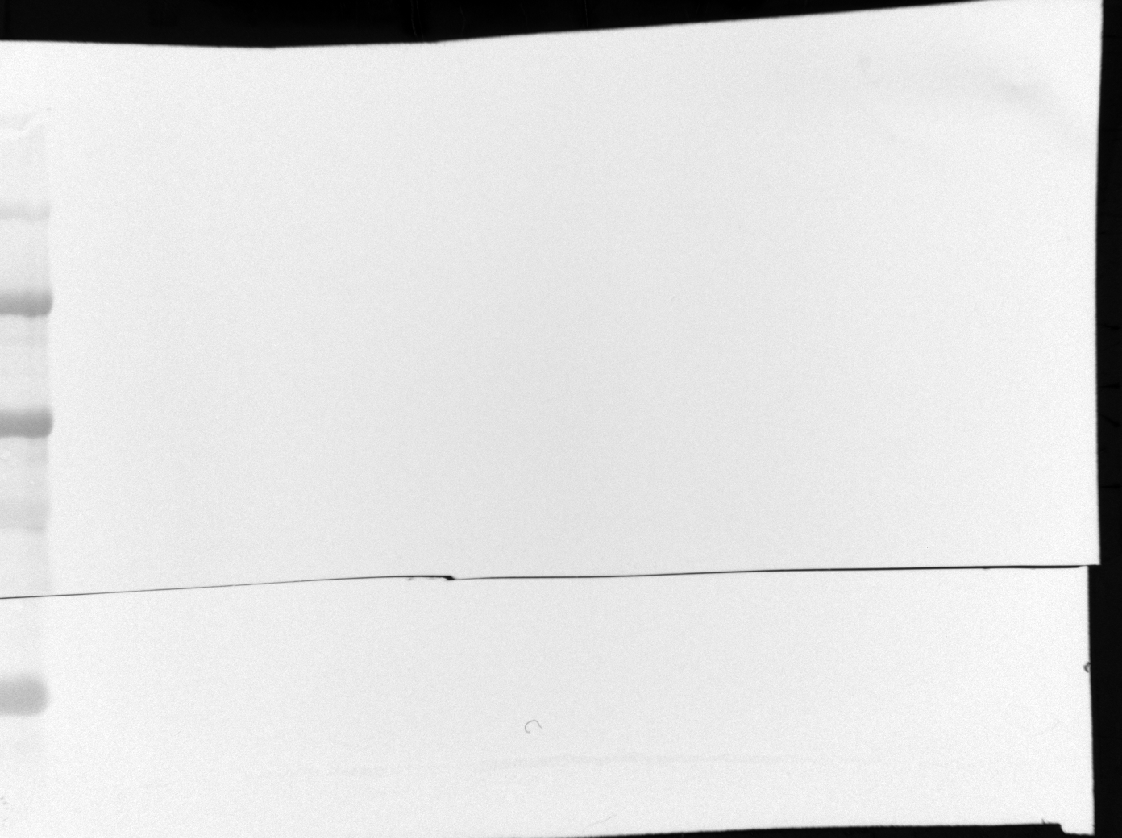

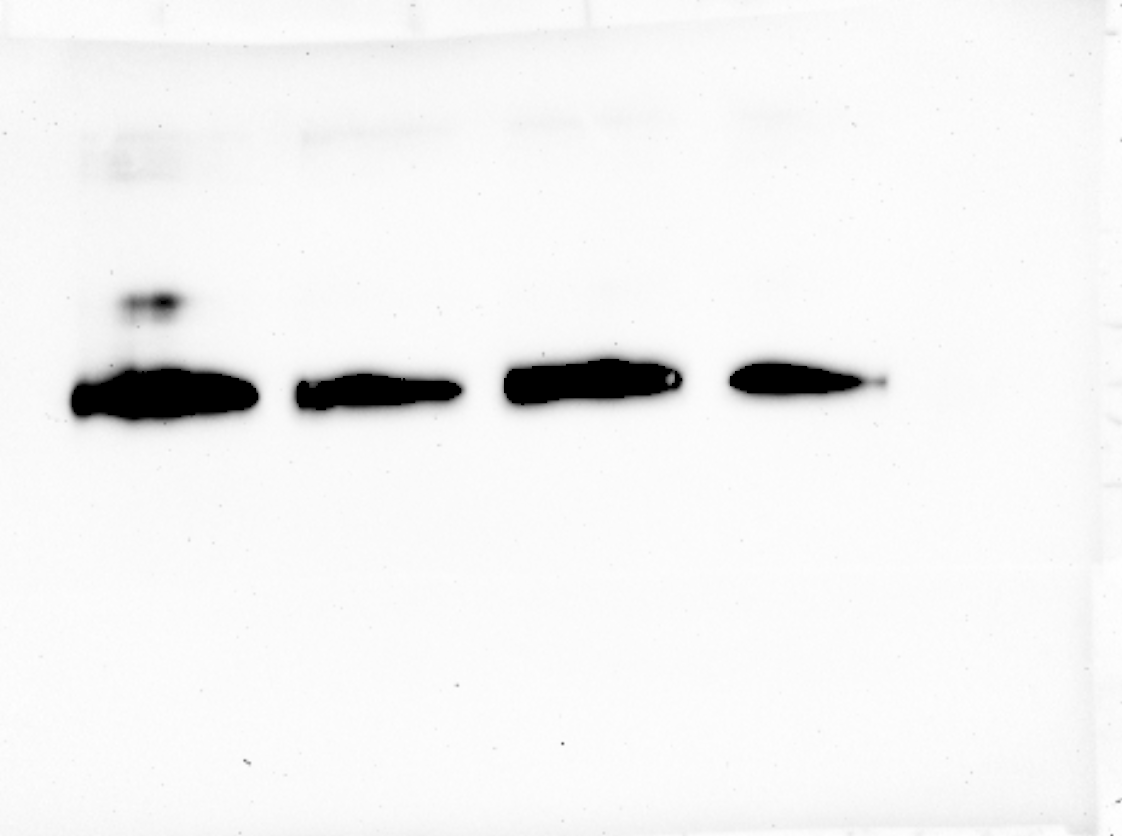

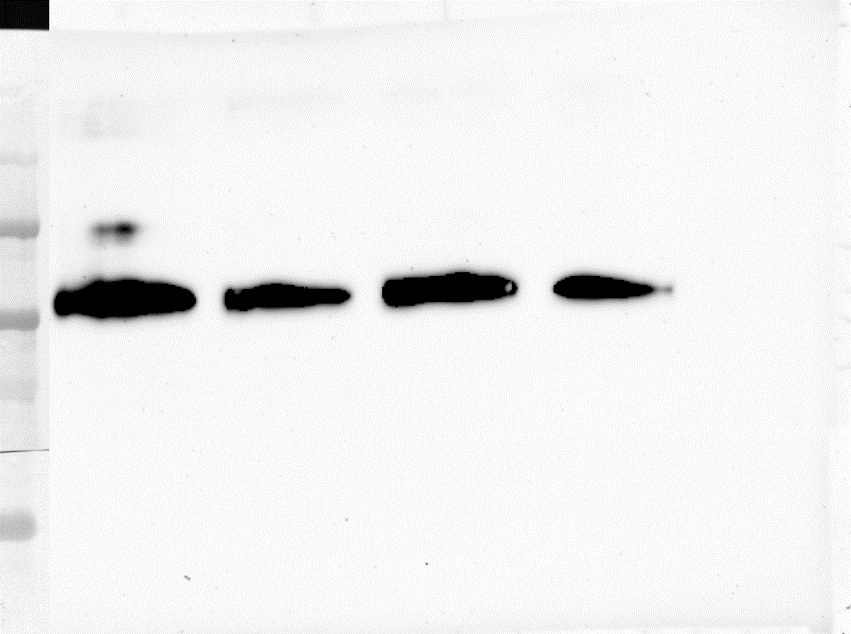


α-KCNQ5

α-actin

250 kDa

150 kDa

100 kDa

75 kDa

50 kDa

37 kDa

WT-P2A-eGFP

R359C-P2A-eGFP

L692V-P2A-eGFP

Q735R-P2A-eGFP

CTRL
