# Supplementary material for: Loss-of-function variants in the KCNQ5 gene are implicated in genetic generalized epilepsies
Source: eBioMedicine. 2022 Sep 9;84:104244. doi: 10.1016/j.ebiom.2022.104244 (PMC9471468; doi:10.1016/j.ebiom.2022.104244)
Supplement: Supplementary file 2 [file mmc2.docx]

**
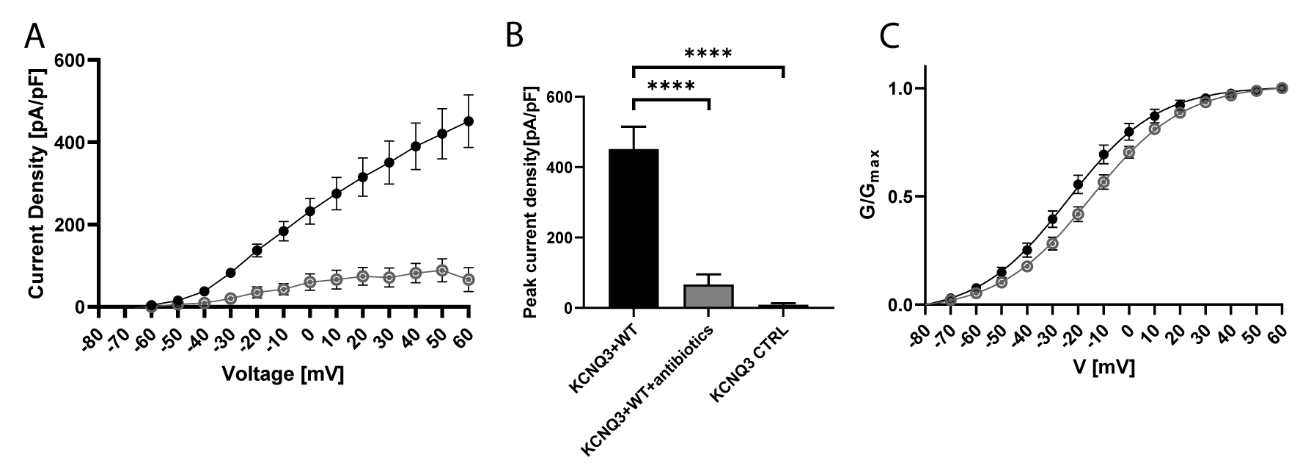
**

**Fig S1. Functional effect of antibiotics in growth media on current density in a K_V_7.3 stable cell line transiently transfected with K_V_7.5.** (**A**) Peak K+ currents normalized by cell capacitances and plotted versus voltage of cells transfected with K_V_7.5-WT (1 µg) in a CHO line stably transfected with K_V_7.3-WT. Black line and dots indicate current density recorded from cells cultured in medium containing zeocin until 72 h prior to recording (n = 13), while the grey line and dots indicate current density recorded in cells cultured in zeocin until recording (n = 6). Cells cultured in medium containing zeocin until recording show a significantly reduced current density as compared to cells where zeocin was removed 72 h prior to recording. (**B**) Comparison of maximum peak current density at +60 mV. (**C**) Voltage-dependent activation curves. Lines represent Boltzmann functions fit to the normalized tail current. Shown are means ± SEM. **** p ≤ 0·0001; **Table S1** provides exact values and statistical analyses.


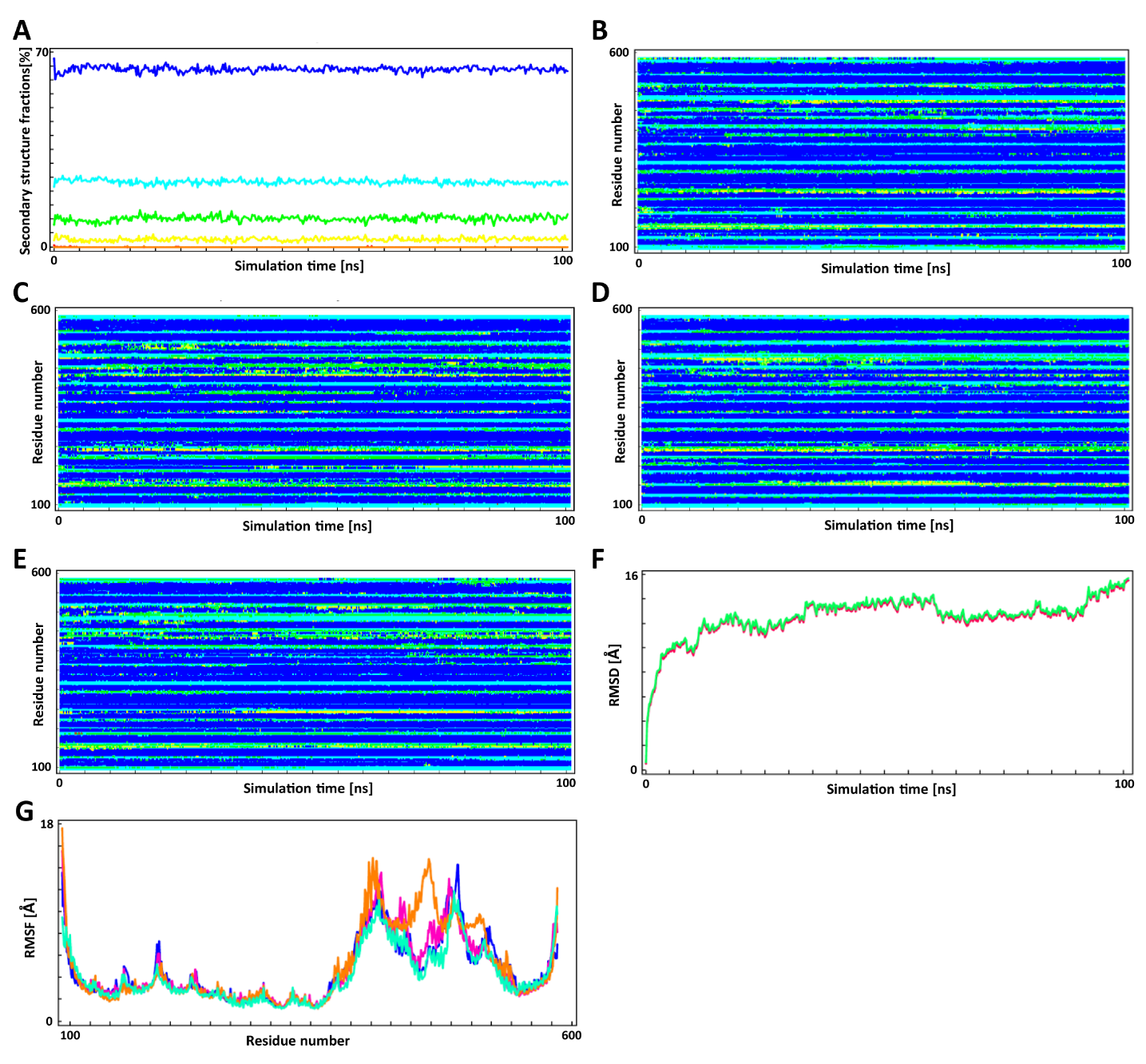


**Fig S2. Simulation 1: K_V_7.5 in PEA.** Molecular dynamic simulations on Kv7.5 (residues 92-586) were conducted. The Kv7.5 represent consensus homology models based on homology to 7VNP.pdb published by Zheng et al., 2022. (**A**) Protein secondary structure content. Blue Helix; red sheet; green turn; turquoise coil; yellow Helix310; orange HelixPi (for **A** to **E**). (**B**) Per-residue protein secondary structure of molecule A. (**C**) Per-residue protein secondary structure of molecule B. (**D**) Per-residue protein secondary structure of molecule C. (**E**) Per-residue protein secondary structure of molecule D. (**F**) Solute RMSD from the starting structure. Blue RMSDCa; red RMSDBb; green RMSDAll. (**G**) Solute protein/nucleic acid residue RMSF. Blue Mol D; pink Mol A; orange Mol B; turquoise Mol C.


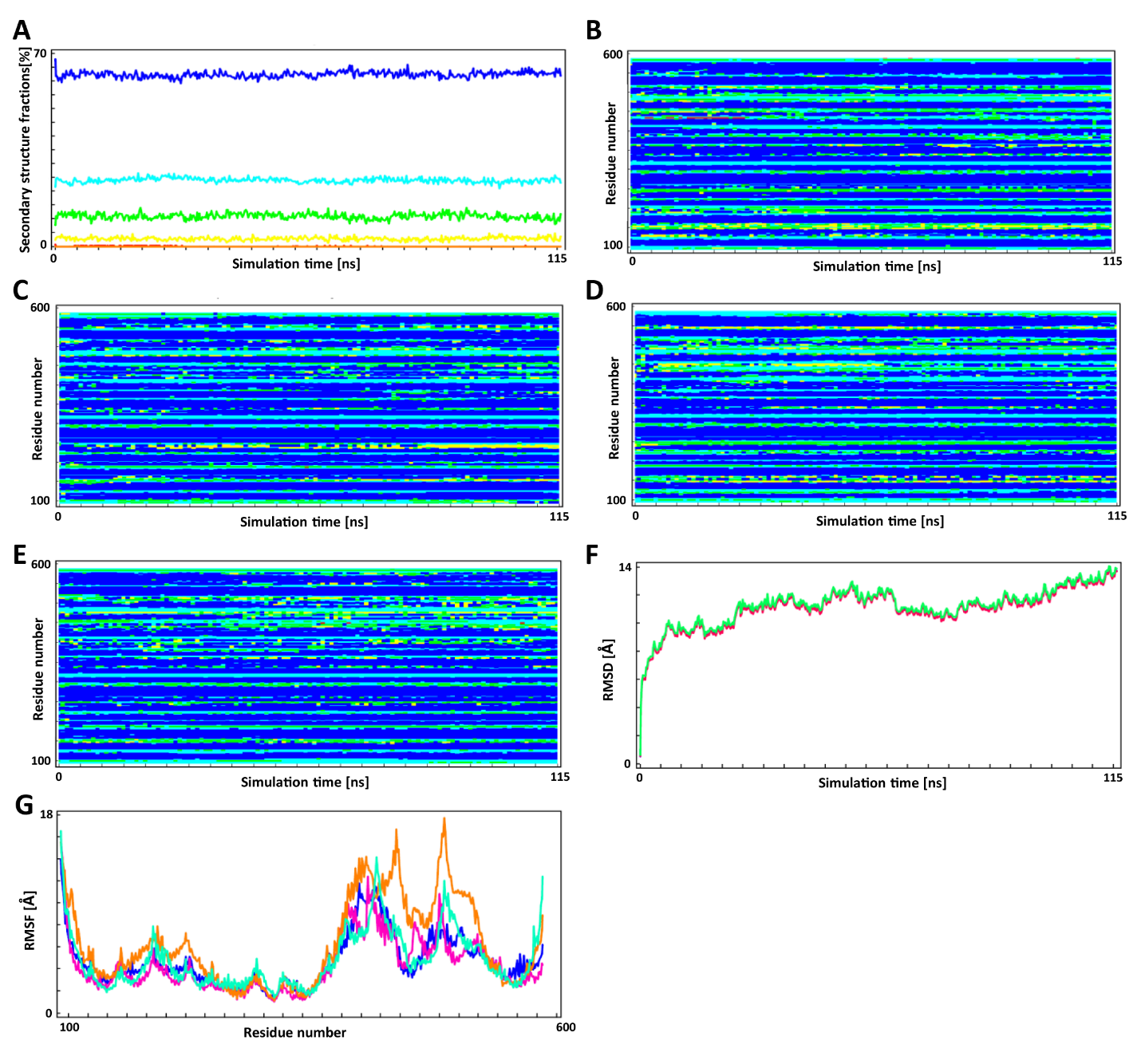


**Fig S3. Simulation 2: K_V_7.5 in PEA.** Molecular dynamic simulations on Kv7.5 (residues 92-586) were conducted. The Kv7.5 represent consensus homology models based on homology to 7VNP.pdb published by Zheng et al., 2022. (**A**) Protein secondary structure content. Blue Helix; red sheet; green turn; turquoise coil; yellow Helix310; orange HelixPi (for **A** to **E**). (**B**) Per-residue protein secondary structure of molecule A. (**C**) Per-residue protein secondary structure of molecule B. (**D**) Per-residue protein secondary structure of molecule C. (**E**) Per-residue protein secondary structure of molecule D. (**F**) Solute RMSD from the starting structure. Blue RMSDCa; red RMSDBb; green RMSDAll. (**G**) Solute protein/nucleic acid residue RMSF. Blue Mol D; pink Mol A; orange Mol B; turquoise Mol C.


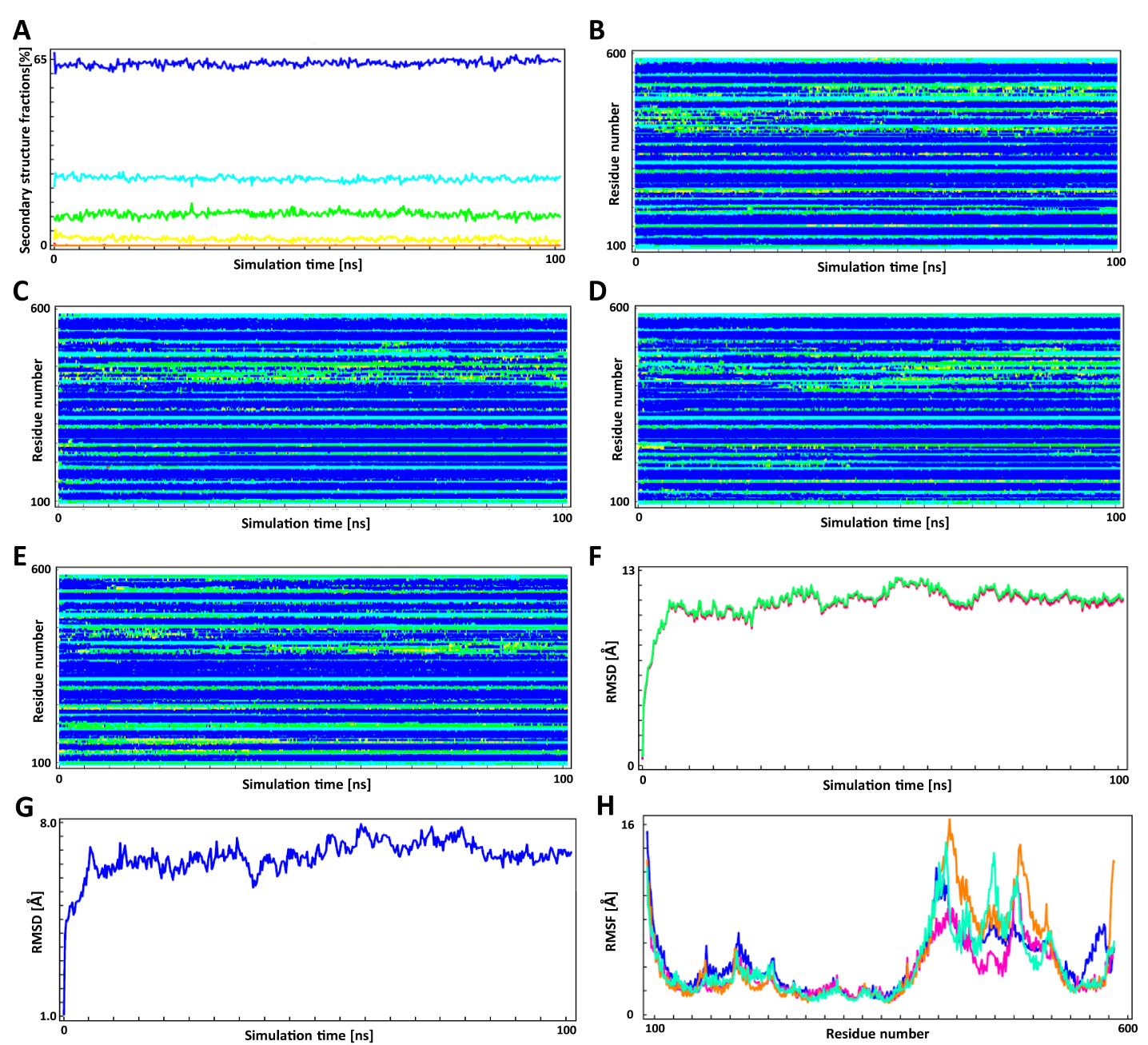


**Fig S4. Simulation 1: K_V_7.5-PIP_2_ in PEA.** Molecular dynamic simulations on Kv7.5 (residues 92-586) were conducted. The Kv7.5 represent consensus homology models based on homology to 7VNP.pdb published by Zheng et al., 2022. In Kv7.5 containing PIP_2_ the lipid was positioned in identical initial position as in 7VNP.pdb and the subsequent Kv7.5-PIP_2_ models were subjected to energy minimizations. (**A**) Protein secondary structure content. Blue Helix; red sheet; green turn; turquoise coil; yellow Helix310; orange HelixPi (for **A** to **E**). (**B**) Per-residue protein secondary structure of molecule A. (**C**) Per-residue protein secondary structure of molecule B. (**D**) Per-residue protein secondary structure of molecule C. (**E**) Per-residue protein secondary structure of molecule D. (**F**) Solute RMSD from the starting structure. Blue RMSDCa; red RMSDBb; green RMSDAll. (**G**) Ligand movement RMSD after superposing on the receptor. Blue RSMDLigMove. (**H**) Solute protein/nucleic acid residue RMSF. Blue Mol D; pink Mol A; orange Mol B; turquoise Mol C.


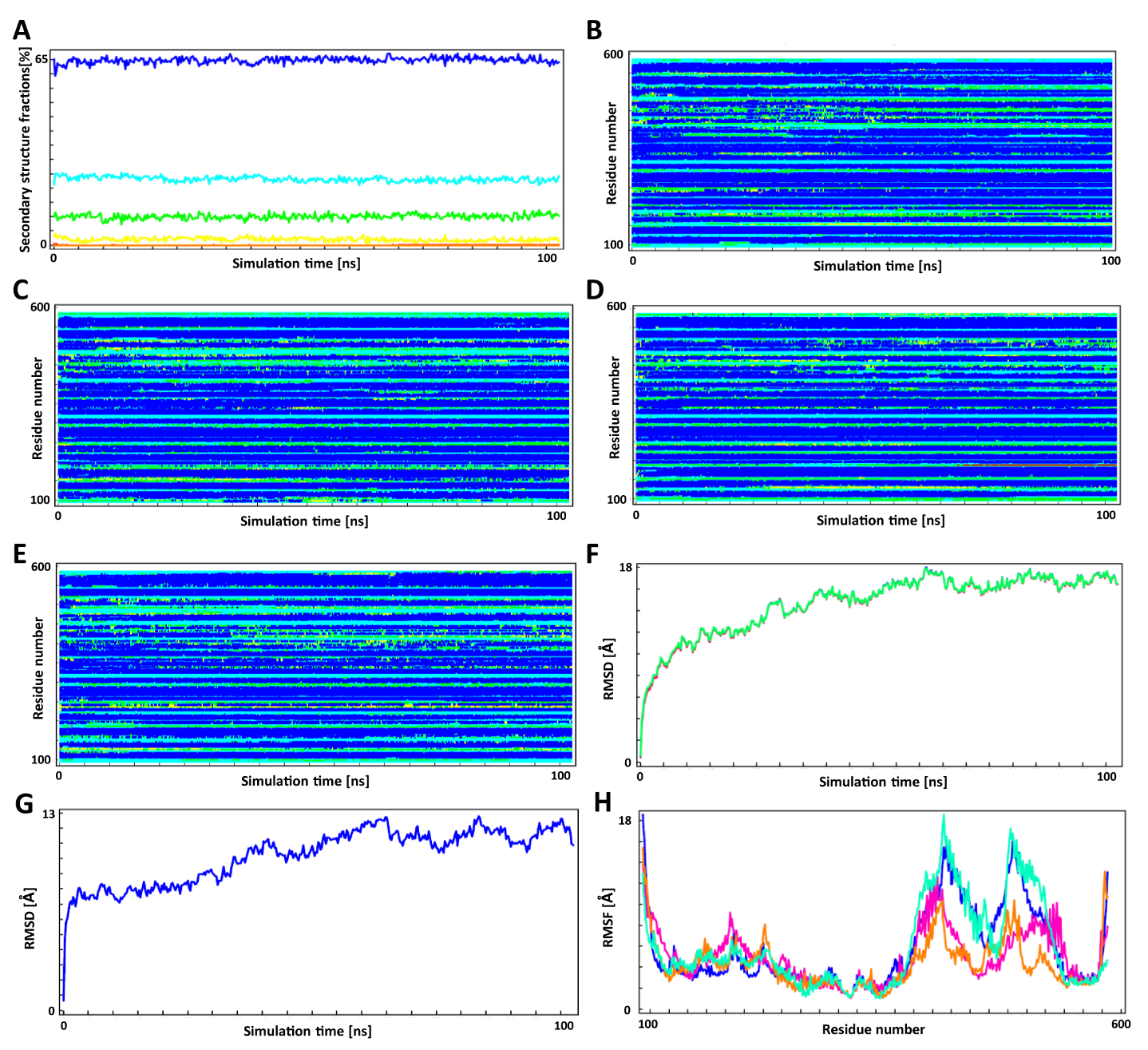


**Fig S5. Simulation 2: K_V_7.5-PIP_2_ in PEA.** Molecular dynamic simulations on Kv7.5 (residues 92-586) were conducted. The Kv7.5 represent consensus homology models based on homology to 7VNP.pdb published by Zheng et al., 2022. In Kv7.5 containing PIP_2_ the lipid was positioned in identical initial position as in 7VNP.pdb and the subsequent Kv7.5-PIP_2_ models were subjected to energy minimizations. (**A**) Protein secondary structure content. Blue Helix; red sheet; green turn; turquoise coil; yellow Helix310; orange HelixPi (for **A** to **E**). (**B**) Per-residue protein secondary structure of molecule A. (**C**) Per-residue protein secondary structure of molecule B. (**D**) Per-residue protein secondary structure of molecule C. (**E**) Per-residue protein secondary structure of molecule D. (**F**) Solute RMSD from the starting structure. Blue RMSDCa; red RMSDBb; green RMSDAll. (**G**) Ligand movement RMSD after superposing on the receptor. Blue RSMDLigMove. (**H**) Solute protein/nucleic acid residue RMSF. Blue Mol D; pink Mol A; orange Mol B; turquoise Mol C.


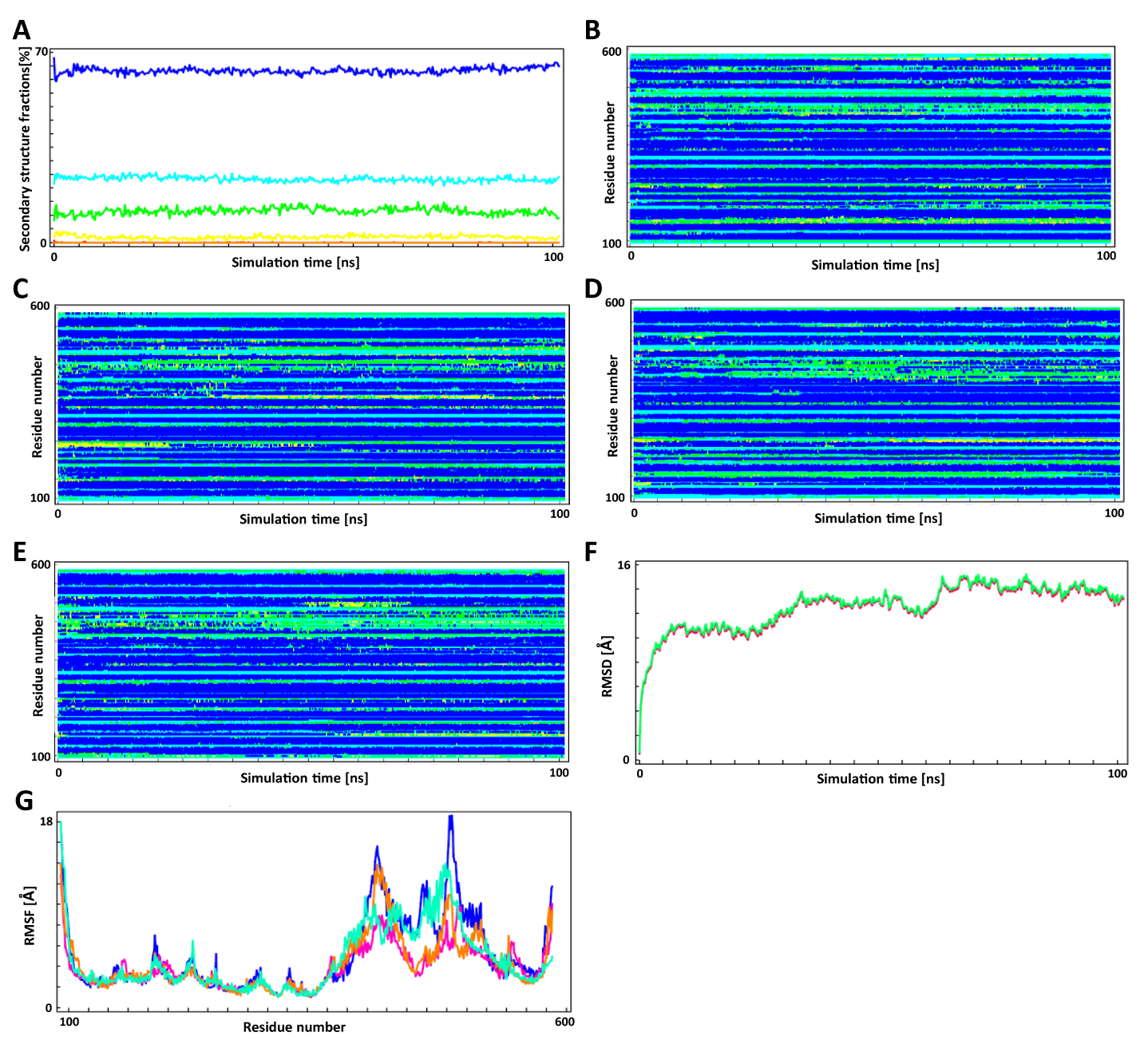


**Fig S6. Simulation 1: K_V_7.5-R359C in PEA.** Molecular dynamic simulations on Kv7.5 (residues 92-586) were conducted. The Kv7.5 represent consensus homology models based on homology to 7VNP.pdb published by Zheng et al., 2022. In Kv7.5 containing PIP_2_ the lipid was positioned in identical initial position as in 7VNP.pdb and the subsequent Kv7.5-PIP_2_ models were subjected to energy minimizations. Kv7.5 R359C models were generated by swapping residues at 359 followed by energy minimizations. (**A**) Protein secondary structure content. Blue Helix; red sheet; green turn; turquoise coil; yellow Helix310; orange HelixPi (for **A** to **E**). (**B**) Per-residue protein secondary structure of molecule A. (**C**) Per-residue protein secondary structure of molecule B. (**D**) Per-residue protein secondary structure of molecule C. (**E**) Per-residue protein secondary structure of molecule D. (**F**) Solute RMSD from the starting structure. Blue RMSDCa; red RMSDBb; green RMSDAll. (**G**) Solute protein/nucleic acid residue RMSF. Blue Mol D; pink Mol A; orange Mol B; turquoise Mol C.


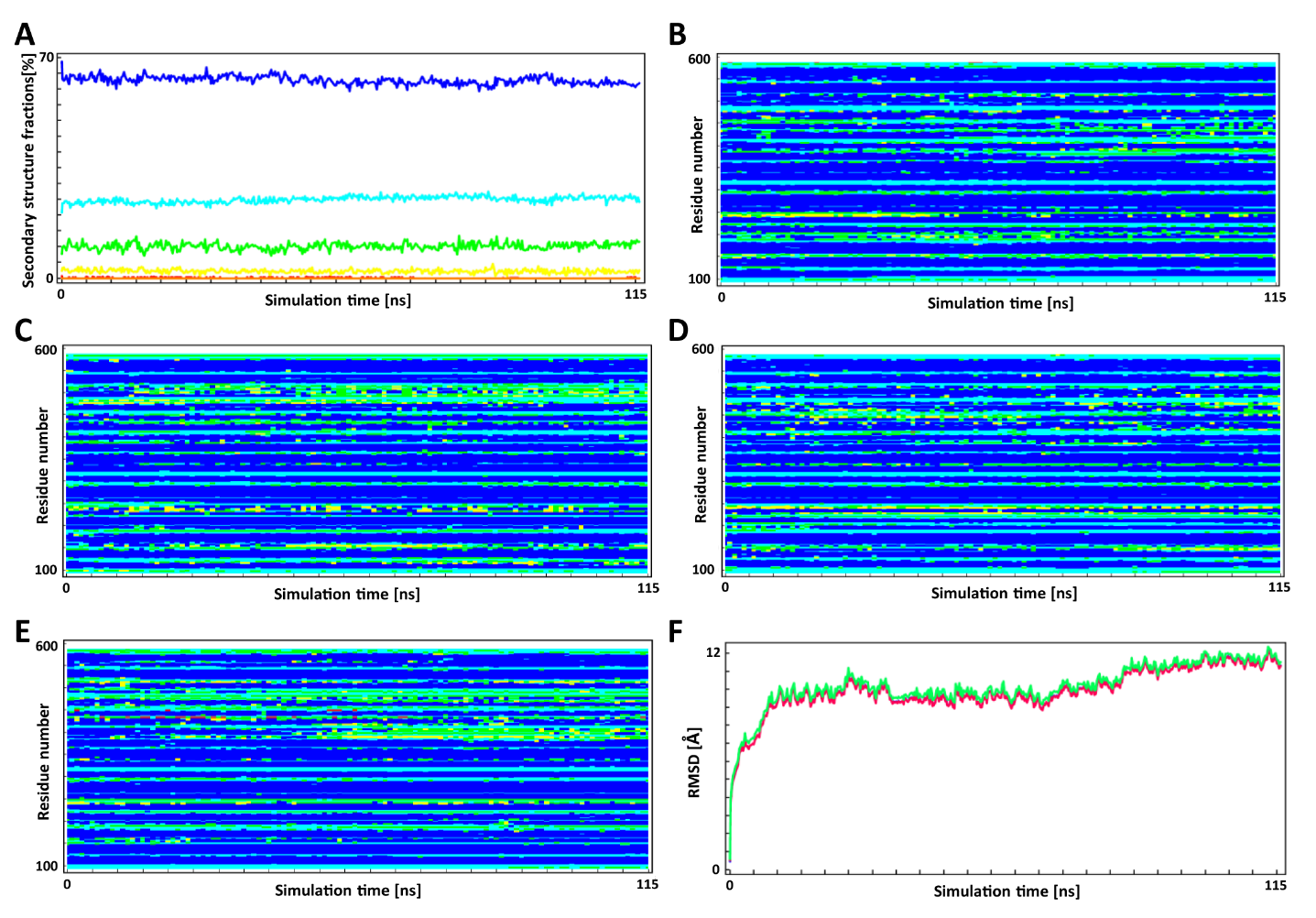


**Fig S7. Simulation 2: K_V_7.5-R359C in PEA.** Molecular dynamic simulations on Kv7.5 (residues 92-586) were conducted. The Kv7.5 represent consensus homology models based on homology to 7VNP.pdb published by Zheng et al., 2022. In Kv7.5 containing PIP_2_ the lipid was positioned in identical initial position as in 7VNP.pdb and the subsequent Kv7.5-PIP_2_ models were subjected to energy minimizations. Kv7.5 R359C models were generated by swapping residues at 359 followed by energy minimizations. (**A**) Protein secondary structure content. Blue Helix; red sheet; green turn; turquoise coil; yellow Helix310; orange HelixPi (for **A** to **E**). (**B**) Per-residue protein secondary structure of molecule A. (**C**) Per-residue protein secondary structure of molecule B. (**D**) Per-residue protein secondary structure of molecule C. (**E**) Per-residue protein secondary structure of molecule D. (**F**) Solute RMSD from the starting structure. Blue RMSDCa; red RMSDBb; green RMSDAll.


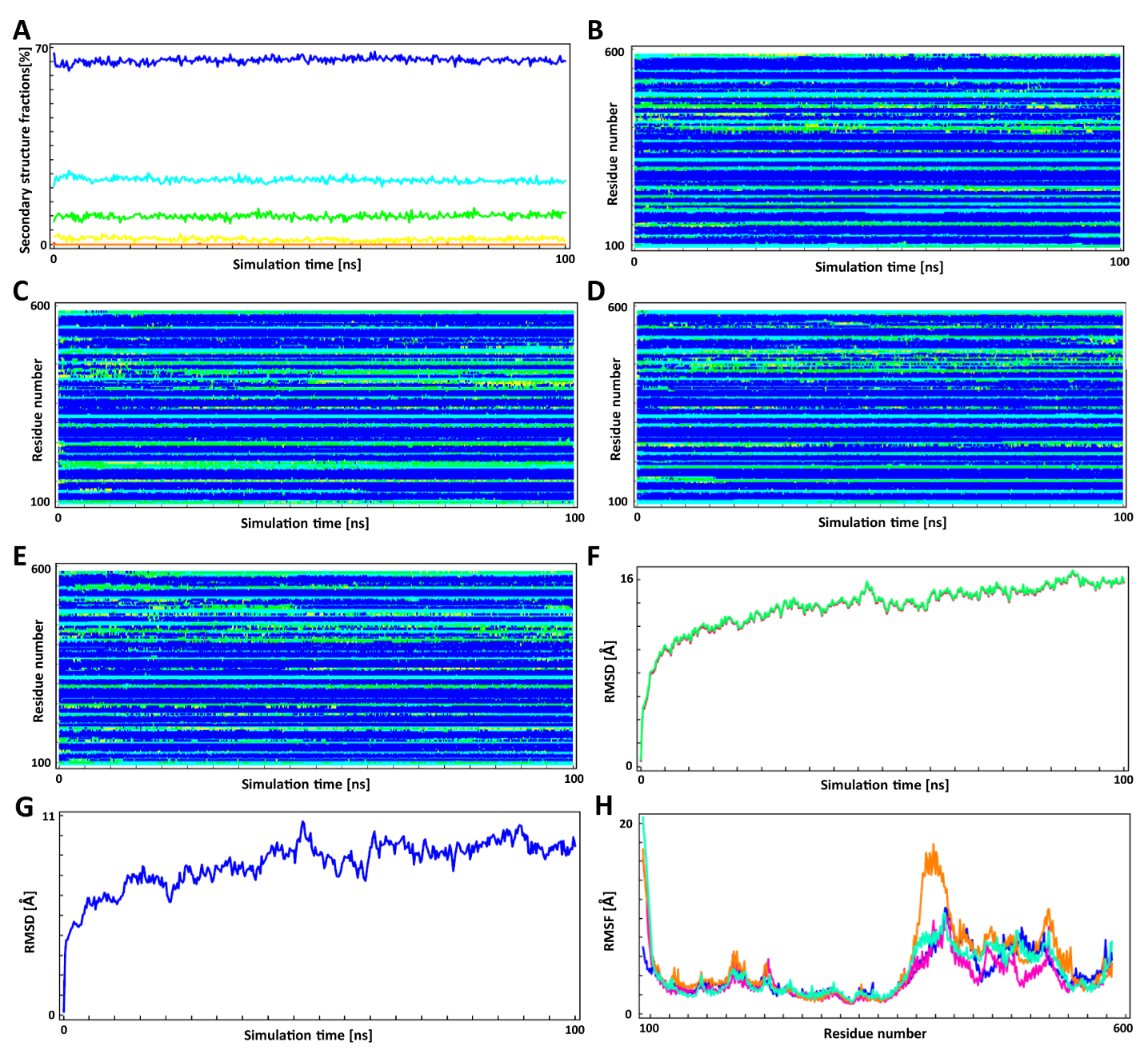


**Fig S8. Simulation 1: K_V_7.5-R359C-PIP_2_ in PEA.** Molecular dynamic simulations on Kv7.5 (residues 92-586) were conducted. The Kv7.5 represent consensus homology models based on homology to 7VNP.pdb published by Zheng et al., 2022. In Kv7.5 containing PIP_2_ the lipid was positioned in identical initial position as in 7VNP.pdb and the subsequent Kv7.5-PIP_2_ models were subjected to energy minimizations. Kv7.5 R359C models were generated by swapping residues at 359 followed by energy minimizations. (**A**) Protein secondary structure content. Blue Helix; red sheet; green turn; turquoise coil; yellow Helix310; orange HelixPi (for **A** to **E**). (**B**) Per-residue protein secondary structure of molecule A. (**C**) Per-residue protein secondary structure of molecule B. (**D**) Per-residue protein secondary structure of molecule C. (**E**) Per-residue protein secondary structure of molecule D. (**F**) Solute RMSD from the starting structure. Blue RMSDCa; red RMSDBb; green RMSDAll. (**G**) Ligand movement RMSD after superposing on the receptor. Blue RSMDLigMove. (**H**) Solute protein/nucleic acid residue RMSF. Blue Mol D; pink Mol A; orange Mol B; turquoise Mol C.


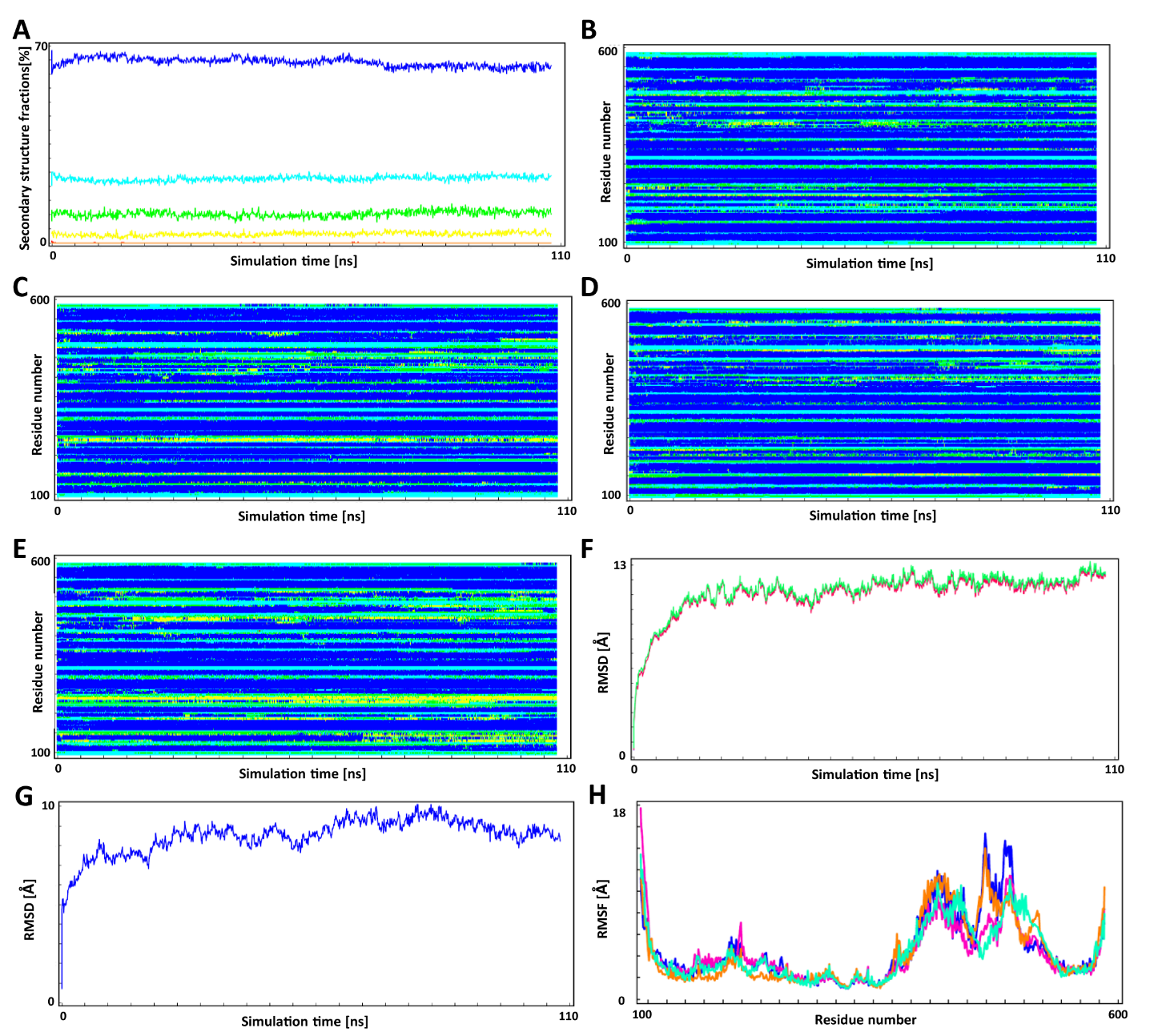


**Fig S9. Simulation 2: K_V_7.5- R359C-PIP_2_ in PEA.** Molecular dynamic simulations on Kv7.5 (residues 92-586) were conducted. The Kv7.5 represent consensus homology models based on homology to 7VNP.pdb published by Zheng et al., 2022. In Kv7.5 containing PIP_2_ the lipid was positioned in identical initial position as in 7VNP.pdb and the subsequent Kv7.5-PIP_2_ models were subjected to energy minimizations. Kv7.5 R359C models were generated by swapping residues at 359 followed by energy minimizations. (**A**) Protein secondary structure content. Blue Helix; red sheet; green turn; turquoise coil; yellow Helix310; orange HelixPi (for **A** to **E**). (**B**) Per-residue protein secondary structure of molecule A. (**C**) Per-residue protein secondary structure of molecule B. (**D**) Per-residue protein secondary structure of molecule C. (**E**) Per-residue protein secondary structure of molecule D. (**F**) Solute RMSD from the starting structure. Blue RMSDCa; red RMSDBb; green RMSDAll. (**G**) Ligand movement RMSD after superposing on the receptor. Blue RSMDLigMove. (**H**) Solute protein/nucleic acid residue RMSF. Blue Mol D; pink Mol A; orange Mol B; turquoise Mol C.
