# Supplementary material for: Loss-of-function variants in the KCNQ5 gene are implicated in genetic generalized epilepsies
Source: eBioMedicine. 2022 Sep 9;84:104244. doi: 10.1016/j.ebiom.2022.104244 (PMC9471468; doi:10.1016/j.ebiom.2022.104244)
Supplement: Supplementary file 3 [file mmc3.docx]

**Table S1. Biophysical properties of K_V_7.5-WT transfected CHO cells cultured without (-) or with (+) Zeocin in F12 medium.**

|  | **- Zeocin** | | | | | **+ Zeocin** | | | | |
| --- | --- | --- | --- | --- | --- | --- | --- | --- | --- | --- |
|  |  | | Activation kinetics | | |  | | Activation kinetics | | |
|  | Current density [pA/pF] | n | V_1/2_  [mV] | k | n | Current density [pA/pF] | n | V_1/2_  [mV] | k | n |
| **WT** | 451·1 ± 44·0 | 13 | -24·05 ± 3·17 | -17·60 ± 2·13 | 13 | 66·34 ± 29·13 ^a^ | 6 | -14·97 ± 2·51 | -18·21 ± 2·66 | 6 |

^a^ *p* < 0·0001 via one-way ANOVA with post hoc correction for multiple comparisons with Dunnett’s test.
